# Sequential accumulation of dynein and its regulatory proteins at the spindle region in the *Caenorhabditis elegans* embryo

**DOI:** 10.1038/s41598-022-15042-8

**Published:** 2022-07-11

**Authors:** Takayuki Torisawa, Akatsuki Kimura

**Affiliations:** 1 Cell Architecture Laboratory, National Institute of Genetics, Mishima, Japan; 2 Department of Genetics, The Graduate University for Advanced Studies, Sokendai, Mishima, Japan

**Keywords:** Mitotic spindle, Motor protein regulation

## Abstract

Cytoplasmic dynein is responsible for various cellular processes during the cell cycle. The mechanism by which its activity is regulated spatially and temporarily inside the cell remains elusive. There are various regulatory proteins of dynein, including dynactin, NDEL1/NUD-2, and LIS1. Characterizing the spatiotemporal localization of regulatory proteins in vivo will aid understanding of the cellular regulation of dynein. Here, we focused on spindle formation in the *Caenorhabditis elegans* early embryo, wherein dynein and its regulatory proteins translocated from the cytoplasm to the spindle region upon nuclear envelope breakdown (NEBD). We found that (i) a limited set of dynein regulatory proteins accumulated in the spindle region, (ii) the spatial localization patterns were distinct among the regulators, and (iii) the regulatory proteins did not accumulate in the spindle region simultaneously but sequentially. Furthermore, the accumulation of NUD-2 was unique among the regulators. NUD-2 started to accumulate before NEBD (pre-NEBD accumulation), and exhibited the highest enrichment compared to the cytoplasmic concentration. Using a protein injection approach, we revealed that the C-terminal helix of NUD-2 was responsible for pre-NEBD accumulation. These findings suggest a fine temporal control of the subcellular localization of regulatory proteins.

## Introduction

Cytoplasmic dynein I is a molecular motor that moves along a microtubule towards its minus-end^1^. Cytoplasmic dynein I is responsible for and plays a vital role in a substantial extent of the minus-end-directed transport in animal cells^2^. In this study, we referred to cytoplasmic dynein I, which consists of multiple subunits, as dynein for simplicity. A striking feature of dynein is that the heavy chain subunit, including ATP hydrolysis sites and a microtubule-binding domain, is encoded by a single gene^3^. This is in contrast to kinesins, which are also recognized as microtubule-based motors that mostly move towards the plus-end of microtubules. In case of kinesins, there are multiple genes encoding kinesin motors that share a similar motor domain; additionally, specific types of kinesin are expressed at specific times and are localized to specific regions to perform specific functions^4,5^. To demonstrate dynein-associated cellular functions, it is imperative that the cell utilizes a single type of dynein heavy chain subunit at various times and locations in a regulated manner. Therefore, the regulation of the localization, timing, and activity of dynein should be sophisticated and should occur at multiple levels, from intramolecular regulation to regulation at the population level^6–8^. An example of intramolecular regulation is an autoinhibition mechanism in which the isolated, solely existing dynein tends to assume a characteristic phi-shaped form and only shows diffusive movements along microtubules^9,10^.

Dynein is associated with various regulatory proteins. Dynactin is a major regulatory protein for dynein. Dynactin associates with dynein at various cellular locations^7^. The formation of dynein-dynactin complex is one of the mechanisms to release dynein from the autoinhibited state, and it provides the basis for the formation of larger complexes with various regulatory proteins^11^. In addition to dynactin, several regulatory proteins, such as LIS1, NDEL1/NDE1, and NuMA exist. LIS1 controls the force generation of dynein and aids formation of the dynein-dynactin complex^12–15^. NDE1/NDEL1 is known to aid the establishment of interaction between LIS1 and dynein^12,16,17^. NuMA recruits dynein to the cell cortex to facilitate the formation of cortical force generators^18^.

Inside the cells, how is the complex formation of dynein with regulatory proteins regulated? When dynein function is needed at a particular location, does dynein translocate to the location after formation of complexes with regulatory proteins, or do the regulatory proteins translocate individually before the complex formation? In most cells, dynein is reserved at the bulk cytoplasm^19–21^. The mechanism and the timing for the formation of the complex remain elusive. A prominent example for considering the spatiotemporal regulation of dynein complexes is the formation of mitotic spindles. Dynein is excluded from the nucleus during interphase, but is incorporated into the spindle during mitosis and plays an important role in spindle formation and function^22^. In spindle formation, nuclear envelope breakdown (NEBD) enables the movement of cytoplasmic molecules into the nuclear region. Dynein and the regulatory proteins involved in spindle formation, such as dynactin, NuMA, LIS1, and NDEL1/NDE1, are translocated into the region^23^. Apart from the proteins related to dynein, spindle component proteins, including tubulin and associated proteins, also present with accumulation in the spindle region.

In the *Caenorhabditis elegans embryo*, tubulin and other molecules, including dynein and its regulatory proteins, were observed to undergo accumulation in the nuclear area after NEBD^24^. Previously, we proposed that tubulin and other molecules could accumulate in the area independent of spindle formation and referred to the accumulating area before spindle formation as ‘nascent spindle region’^24^. The detailed timing and localization of the accumulating protein have not been examined thus far. It has been naively assumed that these proteins translocate to the nascent spindle region simultaneously upon NEBD without specific regulations.

In this study, we analyzed the accumulation of dynein and its regulatory proteins at the spindle region to understand the mechanism by which spatiotemporal regulation of dynein was achieved during spindle formation in the *C. elegans* embryo. Quantitative analysis of the accumulation phenomena showed variations in the amount and the timing of accumulation. Chemical perturbation revealed that the proteins also differed in the accumulation locations within the spindle region, including the spindle microtubules, chromosomes, and/or bulk nucleoplasm. Among the proteins analyzed, NUD-2, a *C. elegans* ortholog of NDEL1/NDE1, showed a characteristic accumulation that started before NEBD. This earlier accumulation process was observed to be dependent on the Ran GTPase activity. The depletion of NUD-2 reduced the ability of the spindle region to retain the accumulated proteins, but it did not affect the accumulation process itself. Furthermore, using the injection technique for the recombinant proteins, we found that the C-terminal helix region of NUD-2 was necessary for its accumulation before NEBD. Our results suggest the implication of the accumulation phenomena for the spatiotemporal regulation of cytoplasmic dynein during the formation of mitotic spindles.

## Results

### Selective accumulation of endogenously tagged dynein and its regulators during spindle formation in *C. elegans* early embryos

To investigate the spatiotemporal regulation of dynein and its regulatory proteins during spindle formation, we observed accumulation events of dynein and its regulatory proteins in *C. elegans* early embryos. Previous studies have shown that dynein and certain regulatory proteins are localized in the spindle region, as evidenced via transgene expression^24,25^. In this study, we used the worm strains expressing the heavy chain of cytoplasmic dynein I (DHC-1), the p150 subunit of dynactin (DNC-1), and several other regulatory proteins, including LIS-1, NUD-2/NDEL1, and LIN-5/NuMA, fused with GFP or mNeonGreen (mNG) from the endogenous locus (Table S1). These proteins were selected because they are involved in mitosis, with presence reported in the cytoplasm during interphase.

**Figure 1:**
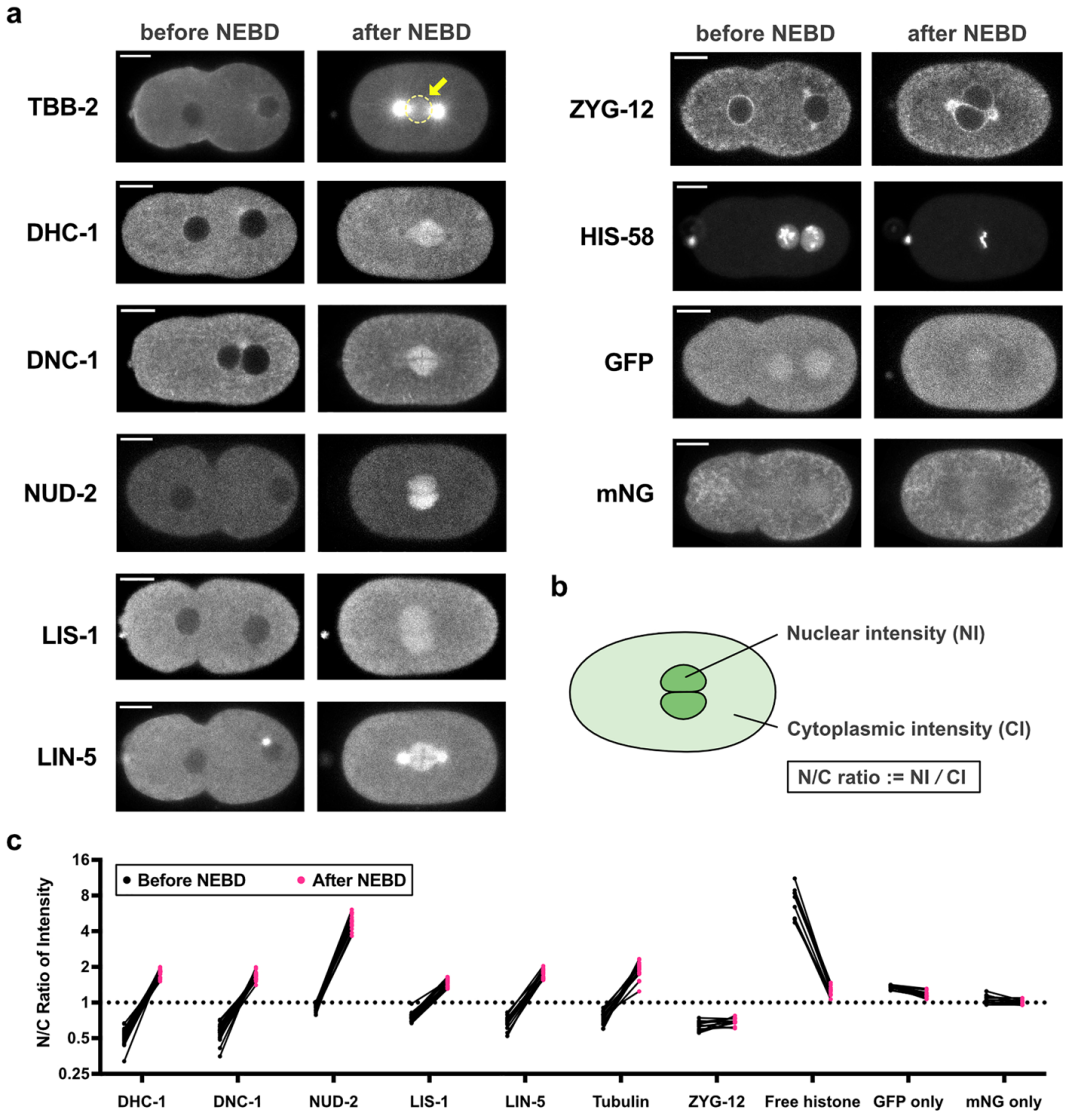
Observations of the temporal dynamics of dynein and its regulatory proteins during the 1st mitosis of *Caenorhabditis elegans* early embryos. (**a**) Typical single-plane time-lapse images showing the cellular localization of tubulin (GFP::TBB-2), dynein (hsGFP::DHC-1), dynactin (mNG::DNC-1), NUD-2 (NUD-2::mNG), LIS-1 (LIS-1::mNG), LIN-5 (LIN-5::mNG), ZYG-12 (GFP::ZYG-12), histone (GFP::HIS-58), GFP, and mNG taken 2 min before and 2 min after NEBD. The yellow circle in TBB-2 indicates the spindle region. Scale bars, 10 µm. (**b**) Schematic representation of quantification of accumulation. Nuclear intensity (NI) and Cytoplasmic intensity (CI) indicate the mean intensity of the nuclear region and cytoplasmic region, respectively. The N/C ratio was calculated with NI and CI measured at the same time point. (**c**) The N/C ratios of dynein and other proteins measured before and after NEBD. Each point represents the N/C ratio before (black) and after (magenta) NEBD. The lines between black and magenta points connect the pairs of individuals. The numbers of pronuclei analyzed are 26 from 14 embryos (dynein, DHC-1), 18 from 11 embryos (dynactin, DNC-1), 19 from 11 embryos (LIS-1), 16 from 8 embryos (NUD-2), 15 from 10 embryos (LIN-5), 16 from 12 embryos (tubulin, TBB-2), 12 from 6 embryos (ZYG-12), 9 from 6 embryos (histone, HIS-58), 10 from 8 embryos (GFP), and 11 from 6 embryos (mNG). The yellow rectangular area indicates dynein and the regulatory proteins focused in this study.

We observed the accumulation of dynein, dynactin, LIN-5, LIS-1, and NUD-2 in the spindle region after NEBD along with tubulin (Figs. 1a and S1a, and Movie S1). We also examined a well-known dynein regulatory protein, Hook (ZYG-12 in *C. elegans*) and found that ZYG-12 did not accumulate to spindle regions (Fig. 1a). The result demonstrates that not all dynein regulatory proteins accumulate at the spindle region. As additional negative controls, we examined four other proteins, free histone (HIS-58, but not on the mitotic chromosomes), GFP, mNG, and MEX-5 (Figs. 1a and S1). MEX-5 is a well-known cytosolic diffusible protein in *C. elegans*^26^. As shown in Fig. 1a, free histone, GFP, and mNG accumulated at the nucleus before NEBD and diffused out to the cytoplasm after NEBD (see also Fig. S9b). MEX-5 was first excluded from the nucleus and entered the spindle region after NEBD, but any apparent accumulation was not observed (Fig. S1b and c).

To check the specificity quantitatively, we calculated how the proteins were concentrated in the spindle region using the ratio of nuclear intensity (NI) to cytoplasmic intensity (CI) before and after NEBD (N/C ratio) (Fig. 1b and c). A multiple-comparison statistical test revealed that the N/C ratios of the dynein regulatory proteins were significantly higher than those of control proteins (GFP, mNG, ZYG-12, MEX-5) at 2-min after NEBD (Fig. S1d). These results demonstrate that the accumulation is specific to selected proteins, including dynein and its regulators.

### Quantification of relative amount of dynein and its regulators accumulated in the spindle region

**Figure 2:**
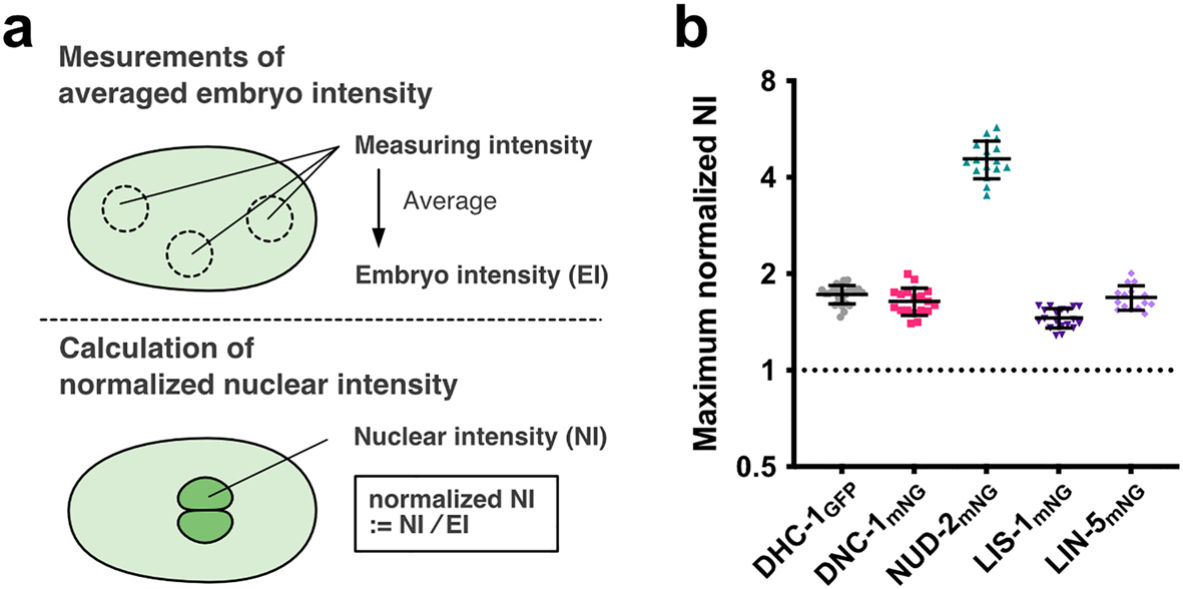
Quantification of accumulated amount of endogenously-tagged dynein and the regulatory proteins. (**a**) Schematic diagram showing the calculation of normalized NIs. The embryo intensity (EI) was measured before pronuclear growth, when the protein distributions are relatively uniform. The EI and maximum NI are shown in Fig. S2. (**b**) The maximum normalized NI (i.e., the maximum NI divided by EI). The analyzed embryos are the same as in Fig. 1. The bars indicate the mean and the SD.

Observations of dynein and regulatory proteins expressed from the endogenous loci enabled quantifying the amounts and stoichiometry of the accumulated proteins. First, we quantified the signal intensity in the cytoplasm before pronuclear formation, when the proteins were distributed uniformly as an index reflecting the total amount of protein inside the cell (“Embryo Intensity (EI)”) (Figs. 2a and S2a). EI varied considerably among the dynein and the regulatory proteins. We then quantified the signal intensity in the spindle region after NEBD. The maximum NIs (maximum NI measured after NEBD; Fig. S2b) also varied among the proteins, and the order was similar to that of the EI with one exception. The NI of NUD-2 increased to a level comparable to that of dynactin (DNC-1), while the EI of NUD-2 was the lowest among the mNG-tagged proteins (Fig. S2). This exception was evident when we calculated the normalized nuclear intensity (normalized NI), the value obtained by dividing the NI by the EI (Fig. 2b). As expected, NUD-2 showed a higher degree of enrichment compared to the other proteins. This suggested the possibility that NUD-2 accumulated with a distinct mechanism from the others to achieve a concentration comparable to that of dynactin. Another interesting feature of the normalized NI, except NUD-2, was that it was almost constant, while the total amount of the proteins (EI) varied. A simple explanation might be that these proteins have a similar affinity for the spindle region, and thus the ratio at the equilibrium is constant.

### Variations in the target locations of accumulations among the proteins

**Figure 3:**
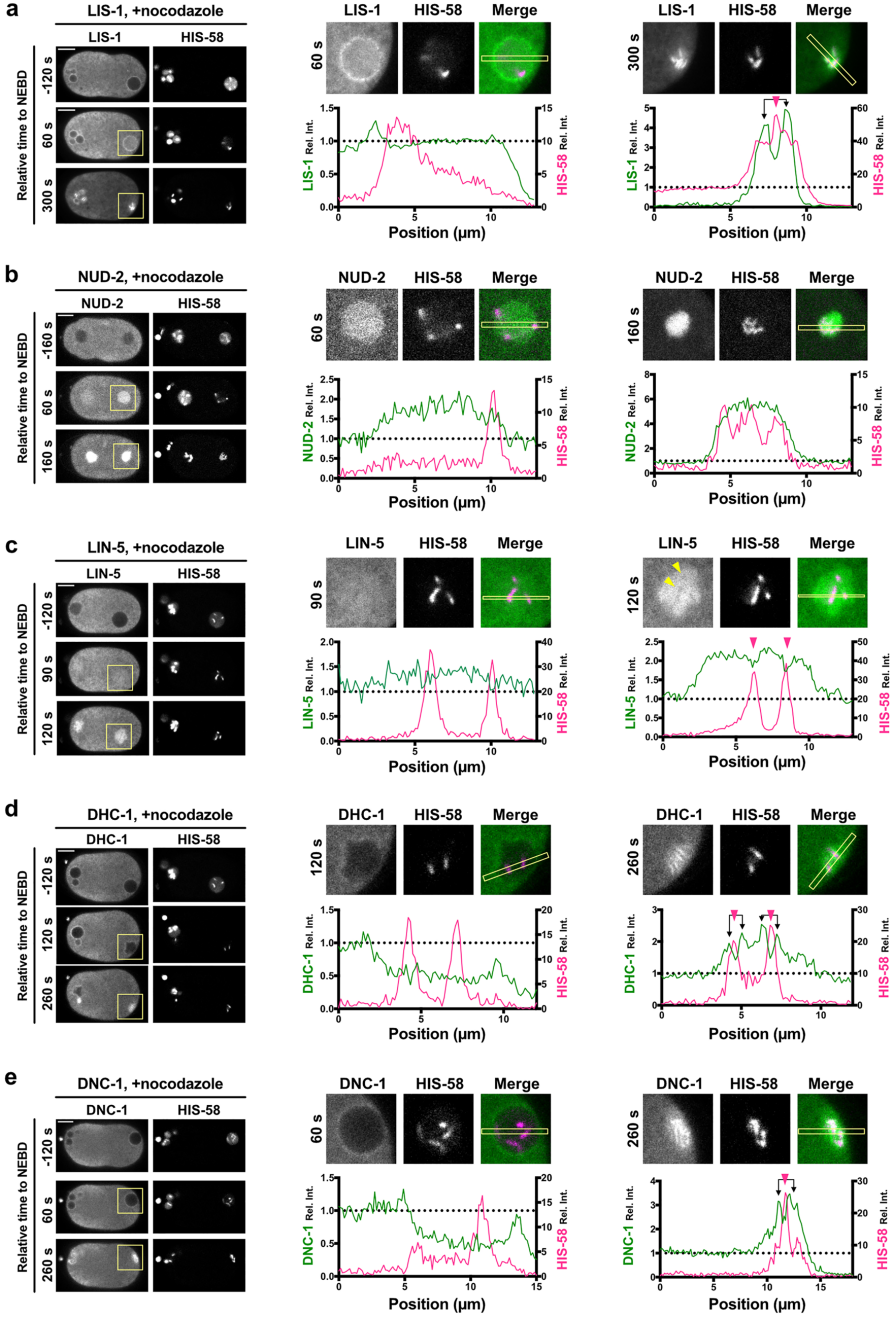
Variations in accumulation locations of dynein and its regulatory proteins. Spatial distribution of LIS-1 (**a**), NUD-2 (**b**), LIN-5 (**c**), dynein (DHC-1, **d**), and dynactin (DNC-1, **e**), in the presence of 10 µg/mL of nocodazole are presented by the single-plane time-lapse images (left), the magnified images (upper center and right), and the intensity profiles (lower center and right) at the indicated timepoint. mCherry-HIS-58 was used to visualize the chromosomes. The timing of NEBD was judged by the decrease of diffusive HIS-58 signals (i.e., free histones) in the nuclear region. Loss of microtubules via nocodazole treatment leads to the defects in pronuclear migration and meeting because these processes are mediated by microtubule-based motors. It results in the delay in NEBD of oocyte pronuclei due to the lack of triggering signal arising from centrosome-associated molecules. The left side of the image corresponds to the anterior. The magnified images have been cropped from the yellow rectangles in the images depicting the corresponding time. Intensity profiles have been calculated in the rectangles indicated in the magnified images. The magenta arrowheads denote the peak of histone signals, and the black arrow lines indicate the signal peak of dynein or the regulatory proteins near the histone peaks. The scale bars indicate 10 µm.

To further investigate the nature of accumulation of dynein and its regulatory proteins, we focused on the spatial distribution of the accumulated proteins. We assumed three candidate locations for accumulation in the spindle regions, namely kinetochores, spindle microtubules, and bulk spindle regions (Fig. S3a). The former two locations are well-known associated regions of dynein and several regulatory proteins^27^. Owing to the abundance of microtubules at the spindle region, it could not be easily ascertained whether a protein was bound to microtubules or whether accumulation occurred at other locations in the spindle region. To eliminate the effects of contribution of microtubules, we used nocodazole treatment and observed the embryos with microtubules depolymerized^24^. In the nocodazole-treated embryos, LIS-1, NUD-2, and LIN-5 continue to demonstrate evident accumulation around the time of NEBD, an event which has been reported to correspond to the accumulation at the nascent spindle region (Fig. 3a–c and Movie S2). LIS-1 and NUD-2 later accumulated around the chromosomes (Fig. 3a and b), while LIN-5 was excluded from the chromosome region (Fig. 3c, arrowheads). As the *C. elegans* chromosome is holocentric, exhibiting possession of multiple kinetochores along the entire length of the chromosome, the localization of LIS-1 and NUD-2 around the chromosome has been assumed to be associated with kinetochores, which is consistent with previous reports^25,28^.

In contrast, dynein and dynactin did not show apparent accumulation in the bulk spindle region, indicating that the accumulations shown in Fig. 1a were mediated by spindle microtubules. Under the nocodazole-treated condition, late accumulation was observed at the chromosomes (Fig. 3d and e, and Movie S2). This finding indicated that both proteins accumulated in the spindle region through the establishment of interaction with kinetochores and spindle microtubules. These spatial accumulation patterns were also confirmed in the *tbb-2* (RNAi) embryos, where tubulin expression was impaired (Fig. S3b). Our results indicated that the proteins showed a spatial variation in their accumulation, and a few proteins could accumulate independently of spindle microtubules. The variation in the localization among the accumulating protein also supports the accumulation is specific to these proteins but not a non-specific behavior to various molecules. In terms of dynein regulation, the results suggest that accumulation in the nascent spindle region before dynein recruitment may contribute to the efficient formation of the required complex.

### Variations in the timing of accumulations

**Figure 4:**
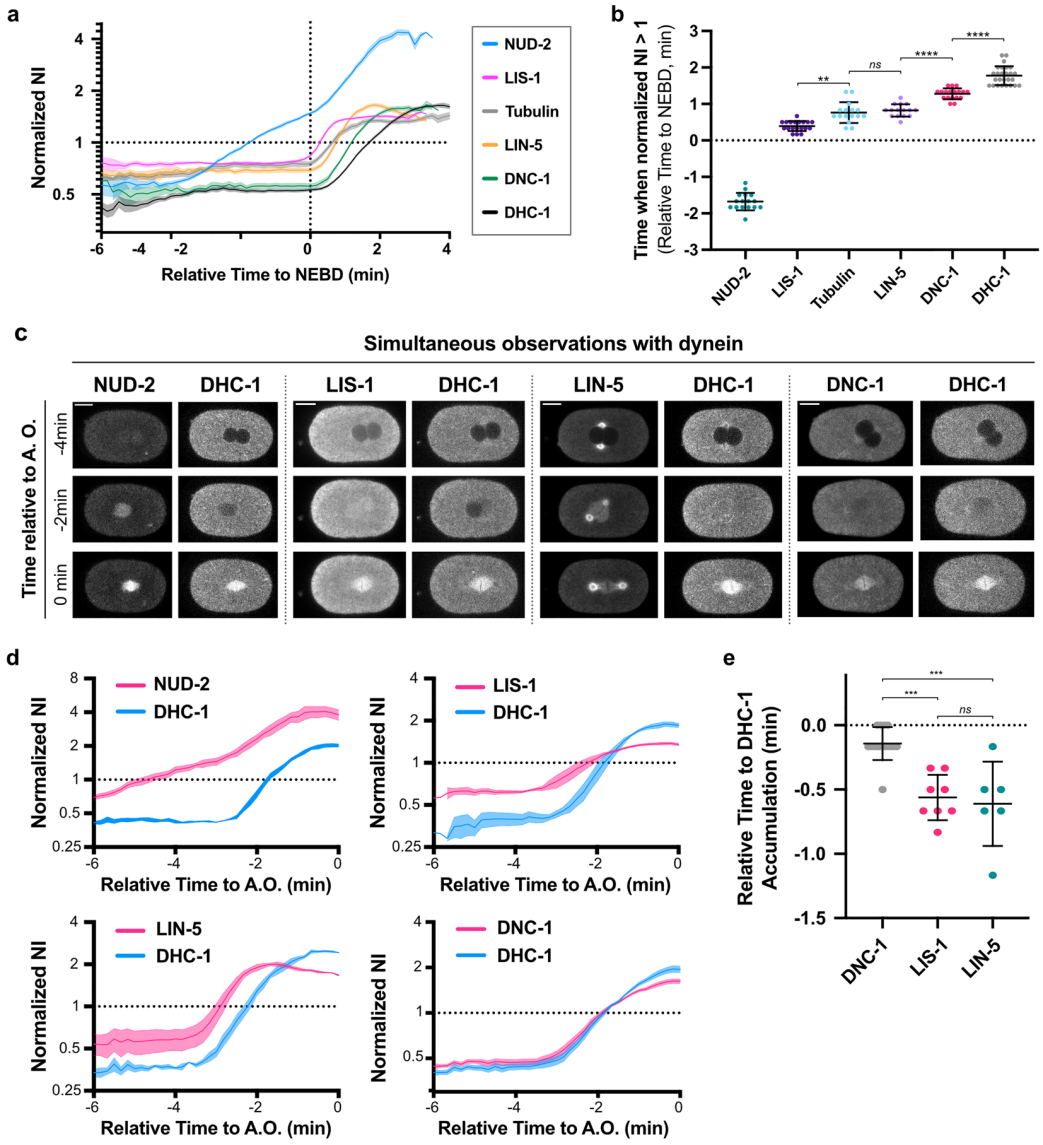
Temporal variations in the accumulation. (**a**) The time series of normalized NI. The initiation time of NEBD, the time origin, has been determined using the intensity decay of free histone (HIS-58) in the nucleus. The numbers of nuclei analyzed were 16 from 8 embryos (NUD-2), 19 from 11 embryos (LIS-1), 16 from 12 embryos (tubulin, TBB-2), 15 from 10 embryos (LIN-5), 18 from 11 embryos (dynactin, DNC-1), and 26 from 14 embryos (dynein, DHC-1). (**b**) The time when the normalized NI exceeds 1, relative to NEBD are shown. The embryos analyzed are the same as in (**a**). (**c** and **d**) Typical single-plane time-lapse images (**c**) and time series of the normalized NI (**d**) were obtained via the simultaneous observations of dynein with LIS-1, LIN-5, NUD-2, and DNC-1. (**c**) The right side of the image corresponds to the anterior. The scale bars indicate 10 μm. (**d**) The plots show the time series of normalized NI obtained from the simultaneous observations exemplified in (**c**). The data quantified for the sperm-derived pronuclei are shown. See Fig. S5b for the oocyte-derived pronuclei. The numbers of pronuclei analyzed are 4 from 4 embryos (LIS-1), 3 from 3 embryos (LIN-5), 4 from 4 embryos (NUD-2), and 8 from 8 embryos. “A. O.” indicates anaphase onset. The mean and SEM are plotted. (**e**) Time difference between the accumulation of DHC-1 and other proteins (LIS-1, LIN-5, and NUD-2). The definition of the accumulation timing is the same as in (**b**). (**c**–**e**) In the experiments where mCherry-tagged DHC-1 was visualized simultaneously with mNG-tagged dynein-regulatory proteins, the timing of NEBD could not be defined as the experiments in (**a**) or others in this study because mCherry-tagged histone do not exist in the strains. Instead of NEBD, the timing of anaphase onset (“A.O.”) was used to normalize the time among the observations. The timing of A.O. was defined as the timing when the segregation of sister chromatids (visualized by the fluorescent signal of DHC-1) was detected for the first time. (**b**, **e**) The statistical significance was determined by Tukey’s multiple comparison test and comparisons between pairs of interest are shown (*ns*: not significant (*p* ≧ 0.05), **: *p* < 0.01, ***: *p* < 0.001, ****: *p* < 0.0001).

Our previous analyses showed that tubulin accumulated in the nascent spindle region with the occurrence of NEBD and suggested that other proteins could also enter the region upon NEBD^24^. We call the accumulation after NEBD “post-NEBD” accumulation. In the present study, we also observed that NUD-2 entered and accumulated in the nuclear region before NEBD (Fig. 4a). We call the accumulation before NEBD as “pre-NEBD” accumulation. Interestingly, NUD-2 is unique among dynein regulatory proteins because of its pre-NEBD accumulation and its high normalized NI after the accumulation (Fig. 2b). The accumulation mechanism of NUD-2 will be investigated and discussed in detail in later sections. Inspired by the early accumulation events of NUD-2 and the distinct localization pattern in the spindle region (Fig. 3), we investigated the accumulation timing of dynein and the regulatory proteins in detail. We used the normalized NI (i.e. NI/EI), rather than the N/C ratio (i.e. NI/CI) to trace the accumulation process. This was because EI is a robust value reflecting the protein concentration in an embryo, whereas CI needs to be quantified for each time point and might fluctuate depending on the region we used for the quantification. The time series of the normalized NI indicated that these proteins did not accumulate in the spindle region simultaneously (Fig. 4a). NUD-2 accumulated earliest among the proteins, followed by LIS-1 and LIN-5. After the accumulation of tubulin, dynein and dynactin accumulated at similar times. This finding was consistent with that of the spatial analysis described above, which suggested that dynein and dynactin accumulated mainly through the establishment of interaction with microtubules. We quantified the accumulation timing using the time (relative to NEBD) when the normalized NI first exceeds 1 (Fig. 4b). A multiple comparison test confirmed the order of accumulation as NUD-2, LIS-1, LIN-5, DNC-1, and DHC-1. We noticed the order of accumulation followed the order of molecular weight (NUD-2 dimer (~ 69 kDa), LIS-1 dimer (92 kDa) and LIN-5 dimer (187 kDa), dynactin (~ 1.0 M), and dynein (1.4 M)), suggesting molecular weights may determine the order. However, this was unlikely. We found that dextran also has some affinity to the spindle region, and dextrans with different molecular weights almost accumulated simultaneously (Fig. S4, Movie S3, and Supplementary Results).

The analyses so far used the different strains, each expressing the single fluorescently-tagged dynein or the regulatory proteins. Therefore, the timings of the accumulation were not compared directly. Meanwhile, we noticed the duration of cell cycle differs among the strains expressing different tagged proteins (Fig. S5a). To further investigate whether the timing of accumulation of the dynein-regulatory proteins relative to DHC-1, we conducted simultaneous observations of dynein and the regulatory proteins by constructing the strains expressing the two fluorescently labeled proteins simultaneously (Figs. 4c, d, S5b and Table S1). We quantified the time when the normalized NI of the regulatory proteins first exceeds 1, relative to the time of DHC-1 (Fig. 4e). Although DHC-1 and DNC-1 appeared to accumulate simultaneously (Figs. 4c, d and S5b), the timing of the DNC-1 accumulation was earlier than that of DHC-1 in a statistically significant manner. Based on the statistical test, we concluded that DNC-1 accumulates slightly earlier than DHC-1. In addition, DHC-1 and DNC-1 displayed a different pattern around the time of saturation; the accumulation speed decreased earlier for DNC-1 compared to DHC-1 (Fig. 4d). These results suggest that at least a certain proportion of the accumulated dynein did not form a complex with dynactin. NUD-2, LIS-1, and LIN-5 were confirmed to accumulate earlier than DHC-1 (Fig. 4e). Of note, the timing of LIS-1 and LIN-5 accumulation relative to the DHC-1 accumulation were not significantly different (Fig. 4e). For the order of LIS-1 and LIN-5, we do not conclude in the present study. In conclusion, the order of accumulation into the spindle region was NUD-2, LIS-1 and LIN-5, DNC-1, and then DHC-1, from the earliest among dynein and the regulatory proteins.

### The spindle region requires NUD-2 to retain the accumulated proteins

In this study, we found pre-NEBD accumulation of NUD-2, which was unique to this protein (Fig. 4a). The pre-NEBD accumulation was also observed in the later stage embryos (2, 4, 8, 16-cell stages), and the onset of the NUD-2 accumulation was independent of the timing of NEBD (Fig. S6, Movies S4, and Supplementary Results). Ran GTPase is a major regulator for nuclear transport^29^. The post-NEBD accumulation of the tubulin molecules in the *C. elegans* embryo is dependent on RAN-1, the *C. elegans* Ran GTPase^24^. Our *ran-1* knockdown experiment indicated that pre-NEBD accumulation of NUD-2 requires RAN-1 (Fig. S7). In contrast to the pre-NEBD accumulation of NUD-2 and to the post-NEBD accumulation of tubulin, the post-NEBD accumulation of NUD-2 did not require RAN-1 (Fig. S7, Movies S4, and Supplementary Results). Therefore, the molecular mechanism of post-NEBD accumulation of NUD-2 should be distinct from that of tubulin and possibly of dynein and other dynein regulatory proteins.

**Figure 5:**
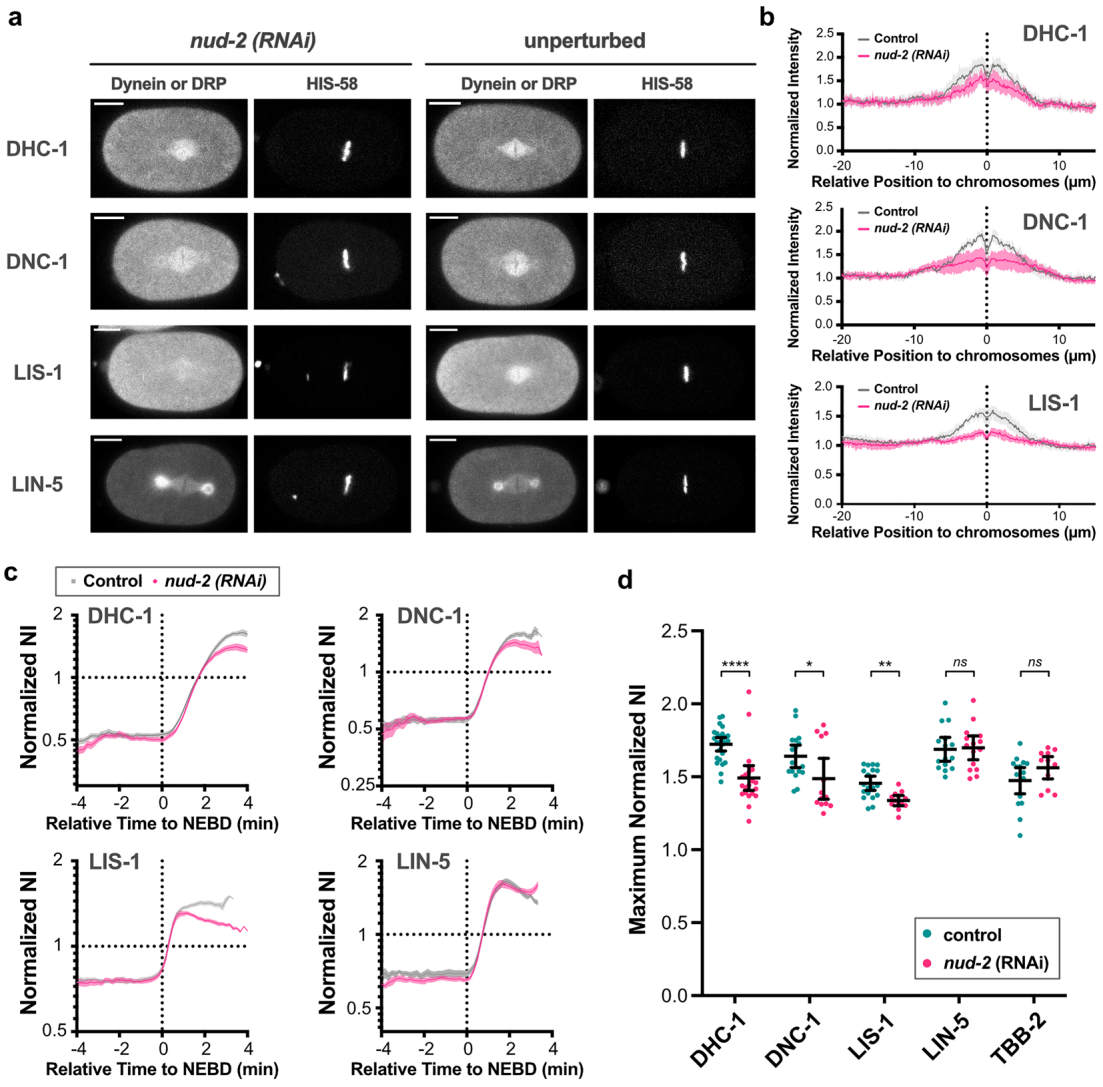
NUD-2 depletion reduces the retained amount of dynein and its regulatory proteins. (**a**) Typical single-plane images of dynein (DHC-1) and the dynein regulatory proteins (DRP: dynactin (DNC-1), LIS-1, and LIN-5) show the comparison between the *nud-2* (RNAi) embryos and the unperturbed embryos. The images of embryos at the anaphase onset were acquired. The left side of the image corresponds to the anterior. The scale bars indicate 10 µm. (**b**) The averaged intensity profiles of dynein (DHC-1, top), dynactin (DNC-1, middle), and LIS-1 (bottom) were derived from both the unperturbed (control) and the *nud-2* (RNAi) embryos. The profiles measured before the anaphase onset are shown. The magenta lines indicate the profiles derived from the *nud-2* (RNAi) embryos, whereas the gray lines indicate the profiles derived from the control embryos. For all analyzed proteins, a reduction in intensity was observed around the chromosomes. (**c**) Temporal dynamics of normalized NI of dynein and its regulatory proteins under the unperturbed or *nud-2* (RNAi) conditions. The gray dots indicate the time series under the unperturbed conditions, whereas the magenta dots indicate the time series in *nud-2* (RNAi) embryos. Before the initiation of a rapid accumulation phase occurred around NEBD, there was no evident difference between the unperturbed and the *nud-2* (RNAi) conditions. The data for the unperturbed condition was the same as shown in Fig. 4a, because the experiments were conducted with the same settings. All analyzed proteins except LIN-5 showed a decay of normalized NI after the rapid accumulation. The numbers of pronuclei analyzed were 22 from 12 embryos (dynein, DHC-1), 13 from 8 embryos (dynactin, DNC-1), 13 from 7 embryos (LIS-1), and 15 from 9 embryos (LIN-5). (**d**) Comparison of the maximum normalized NI between the unperturbed and *nud-2* (RNAi) conditions. Bars indicate mean and SD are shown. Statistical significance was determined by Welch’s *t*-test (***:*p* < 0.001, **: *p* < 0.001, and *: *p* < 0.05) (**b** and **c**) Mean and SEM values are shown.

The earliest accumulation and the highest normalized NI of NUD-2 suggested a role for NUD-2 in the accumulation of other proteins in the spindle region. We observed the accumulation pattern of dynein and the regulatory proteins LIS-1, dynactin, and LIN-5 in *nud-2* (RNAi) embryos. We noticed that their localization level at the spindle was lower than that under the unperturbed condition for all proteins except LIN-5 (Fig. 5a and b). The result is consistent with a previous study that reported the reduction in LIS-1, dynactin, and dynein at the kinetochore in the deletion mutant of *nud-2*^28^. Our present study showed that, in addition to the kinetochore, the reduction also occurred along the entire spindle. In the previous study, an abnormal chromosome behavior was observed in the *nud-2* mutant^28^, which we confirmed using *nud-2* (RNAi) (Fig. S8). These results suggested a causal relationship in which the reduced accumulation of dynein and its regulator caused the spindle defect. However, the defect in spindle function upon the *nud-2* depletion can be a consequence of other processes than the dynein accumulation, such as a reduction in the protein amount of dynein or accumulation of other proteins.

To further investigate the effect of NUD-2 depletion, we analyzed the time series of the normalized NI. The derived time series indicated that the initial accumulation of LIS-1, dynactin, and dynein was not impaired in *nud-2* (RNAi), but the maintenance was defective (Fig. 5c). The result indicated that the protein amount and initial accumulation are normal in *nud-2* (RNAi), thus narrowing down the connection between the accumulation of dynein and the spindle defect. The accumulation was impaired by *nud-2* (RNAi) for dynein (DHC-1), dynactin (DNC-1), and LIS-1, but not for LIN-5 and tubulin, also narrowing down that the defect is associated with these proteins (Fig. 5d). The finding strengthened the idea that the accumulation of dynein, dynactin, and LIS-1 in the spindle region is critical for proper chromosome segregation, and NUD-2 is required to maintain the accumulation.

### The C-terminal helix region of NUD-2 is responsible for pre-NEBD accumulation

**Figure 6:**
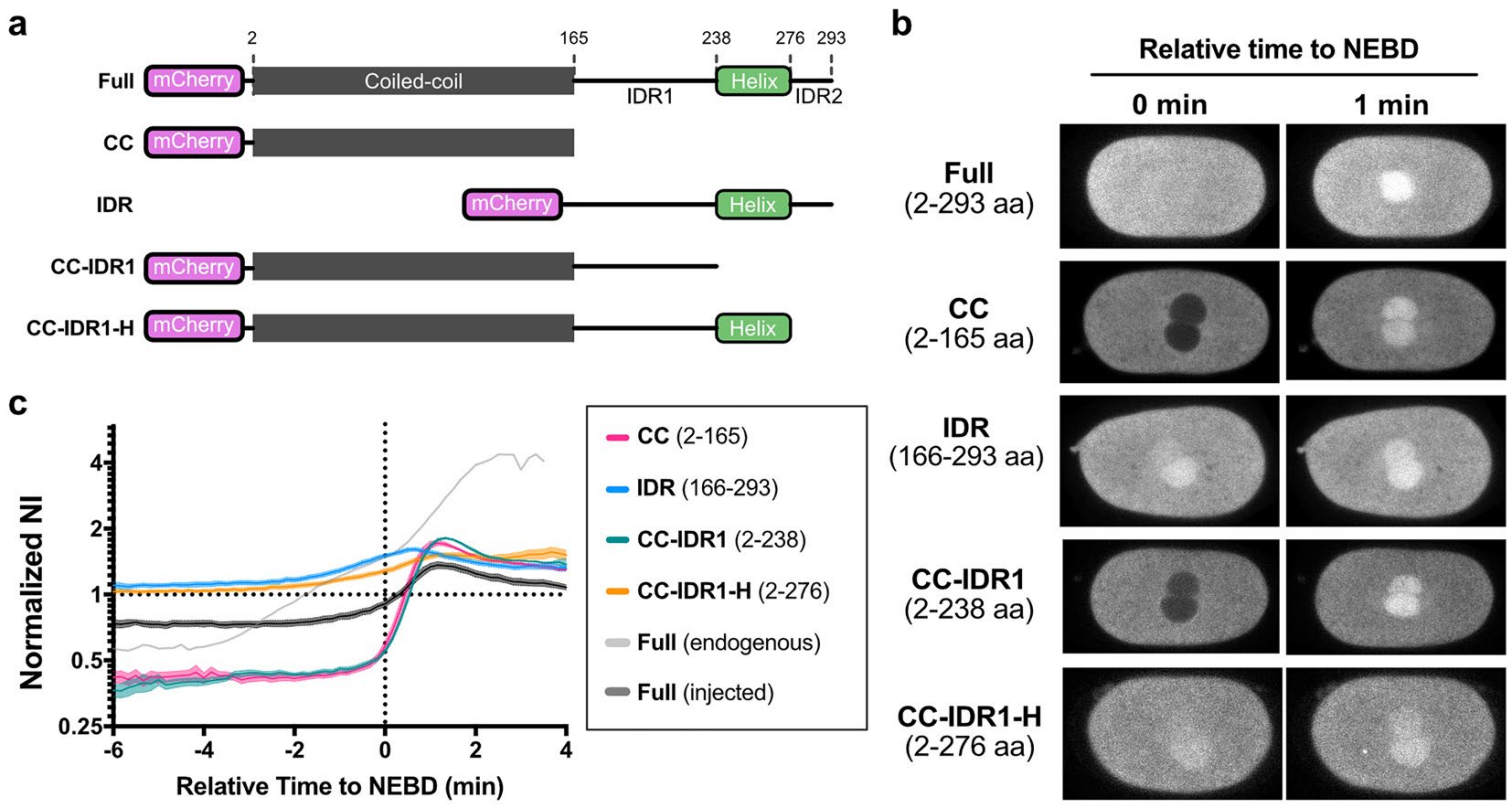
The C-terminal helix of NUD-2 is necessary for pre-NEBD accumulation. (**a**) Schematic representation of the recombinant NUD-2 proteins used for the injection experiments. The fragments were purified from *E. coli* cells. The predicted coiled-coil region is depicted as gray boxes. IDR1 and IDR2 indicate two C-terminal intrinsically disordered regions. Two IDRs flank a C-terminal putative helix region (H) (**b**) Typical single-plane time-lapse images showing the temporal dynamics of the NUD-2 fragments. The left sides of the images correspond to the anterior. The scale bars indicate 10 µm. (**c**) Time series of the normalized NI of the recombinant NUD-2 proteins. The numbers of pronuclei analyzed were 10 from 6 embryos (Full, injected), 9 from 6 embryos (CC, Coiled-coil), 15 from 9 embryos (IDR, IDR1-H-IDR2), 16 from 9 embryos (CC-IDR1), and 10 from 6 embryos (CC-IDR1-H). “Full (endogenous)” is identical to “NUD-2” in Fig. 4a. Mean and SEM values are shown.

Finally, we dissected which region of NUD-2 is responsible for pre-NEBD accumulation of NUD-2. NDEL1/NDE1, the human ortholog of NUD-2, is a homodimeric protein possessing two distinct structural regions. The N-terminal region forms an approximately 20 nm-long coiled-coil structure responsible for the dimerization^30^, whereas the C-terminal region is predicted to be intrinsically disordered. Within the C-terminal region, NDEL1/NDE1 possesses a putative helix region flanked by two intrinsically disordered sequences. The N-terminal region includes one of the two dynein-binding sites and the LIS-1 binding site^30^, aiding binding between LIS-1 and dynein^31,32^. The C-terminal region contains a second dynein-binding site and many phosphorylation sites^33–36^. Based on these structural and functional findings, we investigated the region of NUD-2 necessary for the characteristic pre-NEBD accumulation. For this purpose, we adopted a protein-injection approach. We injected the recombinant NUD-2 fragments into the *C. elegans* gonad and observed the temporal dynamics of the fragments incorporated into the embryos through oogenesis (Fig. 6a, b, and Movie S5). Before observing NUD-2 fragments, we validated our injection method by examining the temporal dynamics of mCherry, which was consistent with that of the transgenic GFP (Fig. S9).

We first observed the accumulation of injected full-length NUD-2. The injected full-length NUD-2 accumulated at the spindle region. Compared to the endogenous protein, the maximum normalized NI was lower, and the accumulation rate was slower for the injected NUD-2. As a result, the normalized NI was below 1 at NEBD (Figs. 4a and 6c). The pre-NEBD accumulation is thus weaker for the injected NUD-2, but exists as we found that the initiation time of accumulation was earlier than that of NEBD. The post-NEBD was evident for the injected NUD-2 as there was a change in the accumulation rate around NEBD. Based on these results, we concluded that the injected protein showed both pre- and post-NEBD accumulation, although the accumulation rate was less than the endogenous one.

We then observed the N-terminal fragment composed of the predicted coiled-coil domain (NUD-2_CC_, 2–165 aa). In contrast to the full-length construct, NUD-2_CC_ was excluded from the pronuclei until NEBD, without pre-NEBD accumulation (Fig. 6b and c). We then observed the C-terminal fragment, which contains two intrinsically disordered regions (IDR1 and IDR2) and a C-terminal putative helix (H) (NUD-2_IDR_, 166–293 aa) and found that NUD-2_IDR_ showed both pre- and post-NEBD accumulations. To further investigate which region was responsible for pre-NEBD accumulation, we next observed NUD-2_CC-IDR1_ (2–238 aa), including the coiled-coil and the first intrinsically disordered region. NUD-2_CC-IDR1_ was excluded from the interphase nucleus and only accumulated after NEBD, which exhibited the same temporal pattern as NUD-2_CC_. Finally, we observed NUD-2_CC-IDR1-H_ (2–276 aa), which contained the region comprising the N-terminal coiled-coil and the C-terminal helix. Interestingly, NUD-2_CC-IDR1-H_ showed both pre- and post-NEBD accumulations. These results suggest that the C-terminal helix region is involved in the pre-NEBD accumulation of NUD-2.

## Discussion

When a molecule demonstrates functions in a cell, it is not always present in the active state. Its association with regulators often aids its activation. In some cases, the localization of each molecule is controlled spatiotemporally, and the complex formation is considered as the rate-limiting process. To understand the cellular regulatory mechanism within a cell, it is necessary to carefully observe the spatiotemporal dynamics of each molecule in vivo and to integrate the available knowledge. Cytoplasmic dynein I (dynein) is a microtubule-based motor that is indispensable for the formation, maintenance, and elongation of mitotic spindles^2^. Recent in vitro studies have revealed the mechanism by which dynein forms complexes with its regulatory proteins and the properties of the complexes^7,11^. Here, we focused on the manner in which dynein localized and functioned at mitotic spindles after NEBD and investigated the spatiotemporal dynamics of dynein and its regulatory proteins using the *C. elegans* early embryos to understand the mechanism of cellular regulation of dynein.

In mitosis, dynein is important to construct spindles^37,38^. Increasing the amount of dynein in the spindle region should promote reactions involving dynein in constructing the spindle. We showed that, in the *nud-*2 (RNAi) embryos, the maximum accumulation level of DHC-1 was reduced (Fig. 5d). Considering the chromosome segregation is defective in *nud-2* knockout^28^ and *nud-2* (RNAi) embryos (Fig. S8), NUD-2-dependent accumulation of dynein at the spindle region is likely important for proper spindle function. It should be noted here that we cannot exclude the possibility that NUD-2 has a dynein-independent role in spindle functions, as shown in other organisms^39^. Further study is needed to clarify the role of the accumulation of dynein and its regulators at the spindle region.

**Figure 7:**
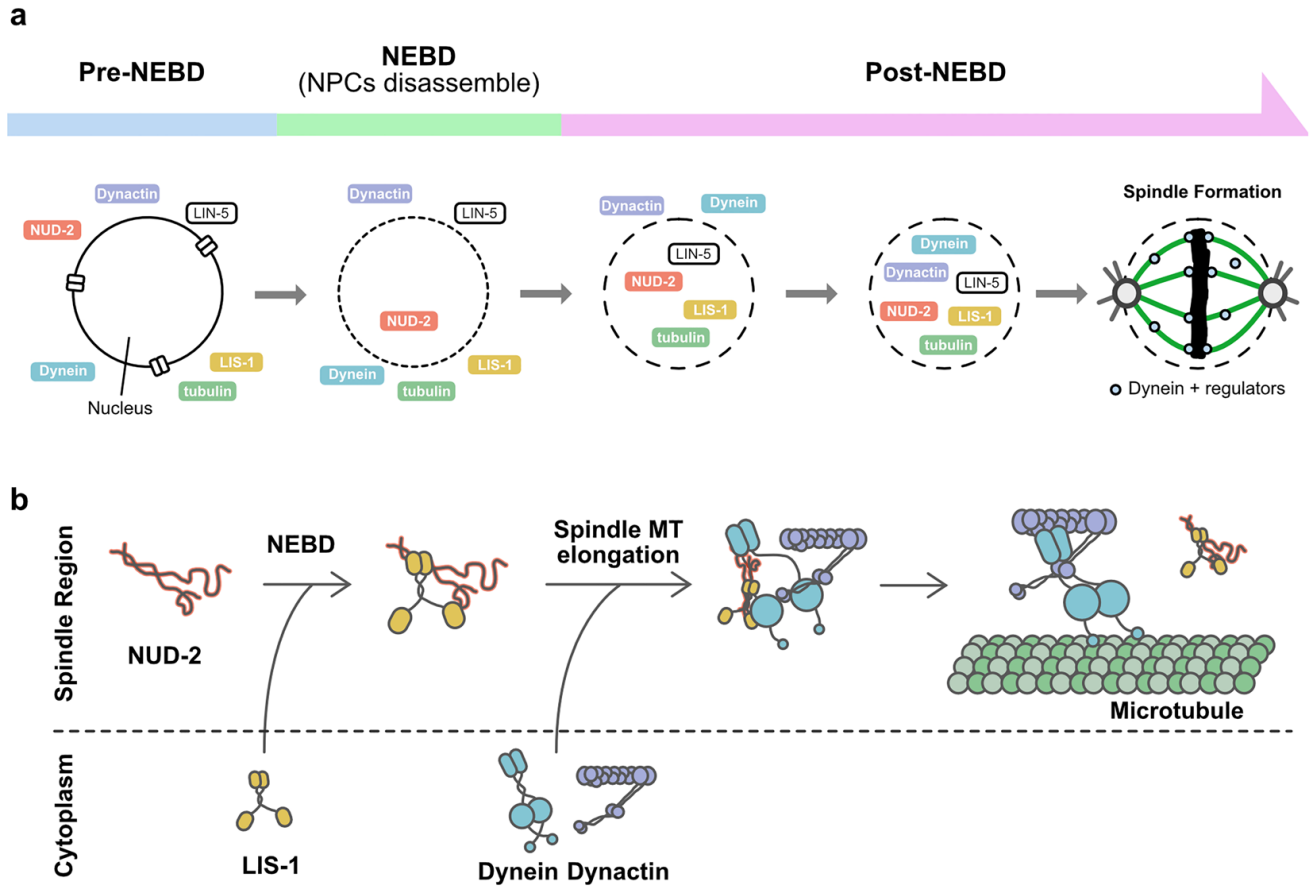
Sequential accumulation of dynein and its regulatory proteins and its implication in dynein regulation. (**a**) Schematic representation showing the sequential accumulation of the proteins observed in this study. NUD-2 accumulates at the spindle region before NEBD. After NEBD, LIS-1 and LIN-5 accumulate, followed by dynein and dynactin. Dynein associates with the accumulated proteins and contributes to the formation and maintenance of mitotic spindles. (**b**) A model of dynein regulation through accumulation. NUD-2, accumulated before NEBD, binds to LIS-1, which accumulates after NEBD. Following the accumulation of LIS-1, dynein and dynactin accumulate. Dynein then associates with LIS-1, which is supported by NUD-2. The binding of LIS-1 changes dynein conformation and facilitates the complex formation of dynein with dynactin. Upon the binding of dynactin to dynein, LIS-1 dissociates from dynein.

By analyzing the time series of normalized NI, we found that dynein and its regulatory proteins did not accumulate simultaneously, but accumulation occurred in a sequential manner (Figs. 4a and 7a). Among the early accumulating proteins, NUD-2 showed the earliest accumulation initiated before NEBD (Fig. 4a). In NUD-2-depleted embryos, we found that dynein, dynactin, and LIS-1 gradually accumulated as in the unperturbed embryos, but the protein concentration in the entire spindle region decreased after the initial accumulation (Fig. 5c). The sequential accumulation and the defects observed for the depletion of the earliest accumulating regulator, NUD-2, suggest a sequential effect, where earlier accumulation affects the dynamics of the proteins accumulated later. In other organisms than *C. elegans*, NUD-2 homolog in *Xenopus laevis* facilitates the accumulation and assembly of lamin-B-containing spindle matrix^40^, suggesting the importance of accumulation for proper cellular processes.

Based on our results, we propose the following scenario for the intracellular regulation of cytoplasmic dynein (Fig. 7b). First, NDEL1, a homolog of NUD-2, recruits LIS1 to dynein^12,32^. If the NUD-2 level decreases in the spindle region, LIS-1 loses one of the interaction partners and leaks out of the spindle region, leading to a reduction in the ratio of dynein bound to LIS-1 in the spindle region. Second, recent studies have shown that LIS1 binding to dynein shifts dynein conformation, promotes dynein-dynactin binding, and dissociates from dynein after the binding of dynactin to dynein^13–15,41^; therefore, a decrease in LIS-1 level decreases the proportion of the dynein-dynactin complex. In both types of dynein complexes, the duration of the presence of dynein on microtubules is expected to be longer^13,15,42,43^. A decrease in the proportion of dynein with a longer duration on microtubules would decrease the affinity for the entire spindle region, leading to a decrease in the maximum accumulation ratio and a decrease in protein concentration at the spindle region after the initial accumulation.

We found that the proteins accumulated earlier than dynein and dynactin were first localized at the bulk spindle region (Fig. 3). This affinity for the bulk spindle region is suggested to enable protein accumulation in the spindle region before the elongation of spindle microtubules. In contrast, dynein and dynactin gradually accumulated through spindle microtubules and localized later to the kinetochores (Fig. 3d and e, black arrows). Our previous research showed that DNC-1 accumulated in the nascent spindle region but not in a uniform manner^24^. Our present study revealed that the accumulation of dynactin was not in the bulk spindle region but rather occurred at the kinetochore. The proteins accumulating in the bulk spindle region increase their absolute concentrations in the spindle region through earlier accumulation, and it may help the efficient complex formation with dynein.

The mechanism to accumulate the molecules in the bulk spindle region is still unclear. Considering the microscopic accumulation process, it is hypothesized that the proteins enter the spindle region through diffusion since it occurs after NPCs are disassembled. If there is a mechanism that entraps the proteins in the spindle region, it results in the accumulation. To investigate the entrapping mechanism, it is notable that accumulation was observed for dextran, which was not expected to interact specifically with intracellular molecules, and such an accumulation was not observed for PEG (Fig. S4c–e). The entrapping mechanism may be related to the molecular properties with a broad specificity, such as charge, hydrophilicity, and molecular structure. The observations with the charged dextrans and PEGs, or other polymers would help us to obtain further insight into the entrapping mechanism. Of course, we cannot exclude the possibility that some factors in the nuclear region after NEBD interact with the accumulating protein through specific protein–protein interactions.

Nuclear accumulation of proteins during mitosis has also been observed in *D. melanogaster*^44–46^ and *A. nidulans*^47,48^. Especially in *A. nidulans*, LIS-1 localizes at the spindle pole earlier than dynein^49^. One characteristic shared by these organisms including *C. elegans* is that their mitosis is semi-open^50^, in which the nuclear envelope disrupts only partially, not completely, during mitosis. It is intriguing to know whether the accumulation of mitotic proteins is universal among the other species. The affinity of the substance for the spindle region of the bulk may differ between cases in which all NEs collapse (open mitosis) and those in which they do not (semi-open or semi-closed).

We assumed that the pre-NEBD accumulation of NUD-2 was related to nucleocytoplasmic transport and NUD-2 dynamics in *ran-1* (RNAi) embryos (Fig. S7a). In RAN-1-depleted embryos, NUD-2 showed only post-NEBD accumulation, suggesting that nucleocytoplasmic transport mediated pre-NEBD accumulation (Fig. S7b and e). We also found that the C-terminal putative helix region was essential for the pre-NEBD accumulation from the protein injection experiment (Fig. 6). Previous studies have shown that the C-terminal region of NDEL1/NDE1 comprises many phosphorylation sites and one of two dynein binding sites, and can bend back onto the N-terminal coiled-coil^17,33–36,50–56^. As suggested by Soares et al. phosphorylation in the C-terminal region might affect the overall molecular structure of NUD-2 by controlling the intramolecular interactions^56^. We propose that the C-terminal region, when not phosphorylated, interacts with the N-terminal region and could not enter into the nucleus. When it is phosphorylated at a certain timing before NEBD, it dissociates with the N-terminal region, which enables NUD-2 to translocate into the nucleus.

As a feature in the methodological aspect, we have injected recombinant proteins into the *C. elegans* gonad, and observed their behaviors in the embryo (Fig. 6). Previous studies (as well as this study, Fig. S4) have injected dextrans to investigate the permeability of nuclear membranes^57,58^. The injection of the proteins widened the application of the gonad injection method. The advantage of protein injection is that the results can be obtained in a shorter time (typically in 1 or 2 weeks) than constructing transgenic worms, which requires the establishment of new worm strains. The behavior of the injected full-length NUD-2 was not identical to the tagged-endogenous protein, as the accumulation rate was less than the endogenous one (Fig. 6c). The reduction in accumulation might be attributed to the saturation of accumulation caused by the presence of endogenous NUD-2 protein.

The accumulation of components required for spindle formation is essential for mitosis, and dynein is a crucial molecule in this process. It will be desirable to study the spatiotemporal dynamics of dynein and its regulatory proteins in species with different modes of mitosis and to compare the mechanisms employed in individual organisms to achieve the universality and diversity of the accumulation phenomenon.

## Materials and methods

### *C. elegans* strains

The worm strains used in this study have been summarized in Table S1 and were maintained at 22 °C on standard nematode growth medium (NGM) plates with OP50 *Escherichia coli*^59^. To establish worm strains expressing both histone and dynein regulatory proteins, we used LP451 (expressing NUD-2::mNG), LP563 (mNG::DNC-1), LP585 (LIN-5::mNG), and LP591 (LIS-1::mNG)^21^, provided by the *Caenorhabditis* Genetics Center. The strains were subjected to crossing experiments with CAL0941 that expressed mCherry-fused HIS-58. CAL2221, which was used to visualize dynein, was established for this study using the CRISPR/Cas9 method^60^. The sequence for the guide RNA was designed to the 5´-terminal of exon1 of *dhc-1* (ATTTTCAGGTAATGGATAG) and inserted to the 5’-terminal of sgRNA scaffold of pDD162 vector^61^. The modified pDD162 vector and the rescued fragment containing full-length *dhc-1* sequence fused with hsGFP were co-injected into the gonads of young adult N2 worms with pRF4, a plasmid used for *rol* mutant screening. hsGFP is a recombinant GFP containing 6xHis-tag and streptavidin-binding peptide tag^62^. After performing screening of the *rol* mutant, the worms were screened using fluorescence signals, and the insertion was confirmed by sequencing. Dynein related processes such as pronuclear migration, spindle elongation, spindle rocking appeared normal suggesting that the fusion proteins of dynein and its regulators are fully functional. For establishing CAL2261, CAL2271, CAL2281, CAL2291, CAL2302, CAL2311, CAL2331, CAL2341, CAL2391, and CAL2461, the corresponding strains listed in Table S1 were crossed. The establishment of the double homozygous mutants was confirmed by fluorescent signal screening. To construct CAL0941 strain, which expresses mCherry::HIS-58 under the control of *pie-1* promoter, we cloned the open reading frame of *his-58* into mCherry_N_GW vector and integrated it into the genome of *unc-119* (ed3) by bombardment as described previously^63^. CAL0234 was constructed using the same procedure as CAL0231^63^.

### RNAi experiments

For the synthesis of double-stranded RNAs (dsRNAs), oligonucleotides containing T3 and T7 promoters were used. The sequences of the oligonucleotides were the same as those available in PhenoBank (https://worm.mpi-cbg.de/phenobank/cgi-bin/ProjectInfoPage.py). The dsRNA sequences were amplified from the genomic DNA of the N2 strain. After amplification, the dsRNAs were synthesized from the products using T3 and T7 RNA polymerases (Promega, P2075, and P2083). The transcription products were incubated at 70 °C for 10 min and at 37 °C for 30 min for annealing. After annealing, the products were filtered using SUPREC™-01 (Takara, 9040). To inject the purified dsRNAs, young adult worms were placed on a thin layer of 2% agarose (Lonza, SeaKem LE agarose) on a 24 × 55-mm coverslip (Matsunami). After covering the worms with halocarbon oil (Sigma, H8898-50MK), the coverslip was mounted and analyzed using an inverted microscope (Axiovert 100, Carl Zeiss). The dsRNAs were injected into the worms using a microinjector (Eppendorf, FemtoJet). After the completion of injection, 5–10 µL of M9 buffer (22 mM KH_2_PO_4_, 42 mM Na_2_HPO_4_, and 86 mM NaCl) was added to the oil to recover the worms. The worms were transferred to a new NGM plate with OP50 *E. coli* and were incubated at 22 °C for 44–48 h (*nud-2*), 24–28 h (*tbb-2*), or 16–20 h (*ran-1*) before conducting observations.

### Construction and purification of recombinant proteins

The full-length coding sequence of *nud-2* was amplified from the cDNA of the N2 strain using the KOD One PCR Master Mix (Toyobo, KMM-101). The amplified sequence was inserted into the pET17b vector (Invitrogen) together with the sequence of SBP-mCherry using seamless cloning with the NEBuilder HiFi DNA Assembly Master Mix (New England BioLabs, E2621). To construct the truncated fragments, unnecessary sequences were removed from the full-length constructs using seamless cloning. The plasmids were transformed into Rosetta2 (DE3) competent cells (Novagen, 71397). The recombinant proteins were purified using SBP-tag and StrepTactin Sepharose. *E. coli* cells obtained from 500 mL culture were harvested using centrifugation for 10 min at 4800 rpm (Beckman, Allegra-30XR), and were subjected to freezing in liquid nitrogen. The collected cells were suspended in lysis buffer (50 mM HEPES–KOH, 150 mM NaCl, 1 mM EGTA, 10% (w/v) sucrose, and pH7.2) supplemented with the ProteoGuard EDTA-free protease inhibitor cocktail (Clontech, 635673). The cells in the suspended solution were sonicated using the Q125 sonicator (Qsonica) and the following settings: 60% amplitude, + 4 °C water bath, and 1-s ON/1-s OFF pulses. The total sonication time was 10 min. The homogenized solution was centrifuged at 75,000 rpm for 15 min (Beckman, TL100.3). The supernatant was loaded onto a StrepTactin Sepharose column with a volume of 1 mL, followed by washing with the lysis buffer. The proteins were eluted with the lysis buffer supplemented with 2.5 mM desthiobiotin. Protein concentrations were determined via the Bradford method using the TaKaRa Bradford Protein Assay Kit (Takara Bio, T9310A).

### Gonad injection of recombinant proteins or polymers

Purified proteins or polymers were diluted using 1 × PBS (Takara Bio, T900) and loaded into custom-made microneedles prepared with the P1000IVF micropipette puller (Sutter Instrument). Young adult worms were placed on a thin layer of 2% agarose (Lonza, SeaKem LE agarose) prepared on a 24 × 55-mm coverslip (Matsunami). After covering the worms with halocarbon oil, the coverslips were mounted and analyzed using the Axiovert100 inverted microscope (Carl Zeiss). Protein solutions were injected into the worms using the FemtoJet microinjector (Eppendorf). After the completion of injections, 5–10 µL of M9 buffer was added to release the worms. After the release, the worms were transferred to a new NGM plate and incubated at 22 °C for at least 3 h to incorporate the injected components into the embryos.

### Imaging of *C. elegans* early embryos

Worms were dissected using a 0.75 × egg salt buffer (118 mM NaCl, 40 mM KCl, 3.4 mM CaCl_2_, 3.4 mM MgCl_2_, 5 mM HEPES pH 7.2). The embryos from the dissected worms were mounted in 0.75 × egg salt buffer, which was placed on a 26 × 76-mm custom-made coverslip (Matsunami). To eliminate the effects of deformation, we did not mount a coverslip on the embryos. Egg-mounted coverslips were set to a spinning-disk confocal fluorescent microscope consisting of the IX71 inverted microscope (Olympus) and the CSU-X1 spinning-head (Yokogawa). The microscope was equipped with a 60 × silicone-immersion objective lens (Olympus, UPlanSApo, 60x/1.30Sil) and a 2.0 × intermediate magnification lens. Images were acquired using an EM-CCD (Andor, iXon) managed by the NIS elements software (Nikon) at 10-s intervals. In single-slice acquisition, the exposure time was 180 ms for both the 488-nm and 561-nm channels. To acquire 3D images, a piezo-actuated microscope stage (PI) was used and the acquisition interval was set to 20 s. In the 3D observations shown in Fig. S7, the exposure times were 120 ms for the 488-nm channel and 60 ms for the 561-nm channel. In the observations of nocodazole-treated embryos, worms were dissected using a 0.75 × egg salt buffer supplemented with 10 µg/mL nocodazole (Fujifilm Wako, 140-08531). After dissection, the worms were mounted using the same buffer. The experimental room was air-conditioned, and the temperature was maintained at 21–23 °C. We analyzed the embryos that we confirmed hatching, except for the RNAi-treated embryos, to exclude the effect of phototoxicity.

### Image analysis

To analyze the images of *C. elegans* early embryos, the background intensity was first subtracted from the entire image, and the photobleaching effect was corrected by assuming an exponential model using ImageJ.

To measure NI (Nuclear Intensity), we manually selected the whole nuclear/spindle boundary using the signals of histones or accumulated proteins and measured the mean intensity, except for LIN5, tubulin, and free histone. In the measurement of LIN-5 and tubulin, which presented with strong signals at centrosomes, the centrosomal signals were masked with a circular region with a 30-pixel diameter (4.09 µm). In the measurement of free histone, a region of the nuclear/spindle region was selected manually to avoid the strong signal from chromosomes.

To measure CI (Cytoplasmic Intensity), two rectangular regions besides the spindle region, long sides of which are along the anterior–posterior axis, were selected manually, and the mean intensity was calculated. The size of rectangles was dependent on the shape and size of the embryo (5–10 µm × 20–30 µm).

To measure EI (Embryo Intensity), we selected an image taken before the growth of pronuclei. This was because the distribution of the proteins is uniform at this stage without apparent cellular polarity and strong localizations at centrosomes. The Mean EI was calculated by averaging the mean intensities measured in three circular regions with a 100-pixel diameter (13.6 μm) randomly placed in the embryos (Fig. 2a).

The N/C ratio in Figs. 1 and S1 was calculated as follows. For the measurements of “before NEBD” values, we choose the time point 2 min before NEBD for all the proteins. For the measurements of “after NEBD” values, we choose the time point when the NI of the proteins reached the maximum for DHC-1, DNC-1, NUD-2, LIS-1, LIN-5, and TBB-2. For the other proteins that do not show spindle accumulation, including free histone, MEX-5, GFP, and mNG, we choose the timing when the N/C reached the maximum within the time interval between 2.0 and 3.5 min after NEBD, based on the timing when the NI of dynein and the regulatory proteins reach the maximum value (Fig. S1e). This time window corresponded to the time points when the dynein regulatory proteins reached the maximum (Fig. S1e, Table 1).

**Table 1:** Quantification of the accumulation of dynein and the regulatory proteins expressed from the endogenous locus.

	Embryo intensity (EI)	Maximum nuclear intensity (NI)	Maximum normalized NI	Time that NI reach its maximum (relative to NEBD)	n
	× 10^2^ a. u	× 10^2^ a. u		min	
NUD-2	1.4 ± 0.2	6.3 ± 1.9	4.6 ± 0.6	2.7 ± 0.3	8
LIS-1	9.0 ± 1.7	13.1 ± 2.6	1.5 ± 0.1	2.0 ± 0.5	11
LIN-5	5.9 ± 1.1	10.2 ± 2.3	1.7 ± 0.2	1.8 ± 0.2	10
DNC-1	3.4 ± 0.7	5.7 ± 1.8	1.6 ± 0.2	3.6 ± 0.8	11
DHC-1	2.1 ± 0.6	3.7 ± 1.2	1.7 ± 0.1	3.5 ± 0.6	9

Mean and SD are shown. The number of embryos analyzed are shown in the right column.

The normalized NI was calculated by dividing NI values at each time point by the EI value.

### Statistical analyses

The multiple comparisons of mean values shown in Figs. 2b, 4b, e, S1d, S2a, and b were conducted by Tukey’s test. The differences of the mean maximum NIs of accumulated proteins between the unperturbed and *nud-2* (RNAi) conditions were tested by Welch’s *t*-test^64^. The statistical calculations were performed using Graphpad Prism 9 (Graphpad Software). In presenting the time series of normalized NIs, mean and standard error of mean are shown.

## Supplementary Information

Supplementary Video 1.

Supplementary Video 2.

Supplementary Video 3.

Supplementary Video 4.

Supplementary Video 5.

Supplementary Information 1.

## Data Availability

The datasets used and/or analysed during the current study available from the corresponding author on reasonable request.
