# Supplementary material for: Sequential accumulation of dynein and its regulatory proteins at the spindle region in the Caenorhabditis elegans embryo
Source: Sci Rep. 2022 Jul 11;12:11740. doi: 10.1038/s41598-022-15042-8 (PMC9273622; doi:10.1038/s41598-022-15042-8)
Supplement: Supplementary file 6 — Supplementary Information 1. [file 41598_2022_15042_MOESM6_ESM.pdf]

Supplemental Material for

Sequential accumulation of dynein and its regulatory proteins at the spindle region in the *Caenorhabditis elegans* embryo

Takayuki Torisawa[1, 2] and Akatsuki Kimura[1, 2]

[1]Cell Architecture Laboratory, National Institute of Genetics, Mishima, Japan

[2]Department of Genetics, The Graduate University for Advanced Studies, Sokendai, Mishima, Japan

Supplementary Results

1. Molecular weight is not the determinant of accumulation order

We observed that the timing of accumulation differed between dynein and its regulatory proteins. The proteins were expected to enter the spindle region mainly through diffusion because NPCs, which act as a diffusion barrier and as a mediator for active nucleocytoplasmic transport, underwent disassembly by that time [1]. Thus, we hypothesized that the difference in diffusion rate depending on molecular weight accounted for the temporal difference. This hypothesis was supported by the fact that the accumulation order of dynein-regulatory proteins coincided with the order of molecular weights; NUD-2 dimer (~69 kDa) accumulated first, followed by LIS-1 dimer (92 kDa) and LIN-5 dimer (187 kDa), with final accumulation of dynactin (~1.0 M) and dynein (1.4 M) (Figure 4a). To examine the effect of molecular weight on the accumulation, we observed the temporal dynamics of polymers with defined molecular sizes using an injection method (Figure S4a)[2,3].

Although previous studies have reported the presence of injected dextran in interphase embryos[2,3], it was unclear whether they accumulated at the spindle region during mitosis. Thus, we decided to observe the accumulation events of polyethylene glycol (PEG) as well as dextran. By observing dextran (40 kDa) and PEG (40 kDa) accumulations, we found that dextran showed NEBD-dependent accumulation in the spindle region (Figure S4b), while PEG was excluded from the nucleus throughout the cell cycle (Figures S4c and S4d). Notably, PEG with a smaller molecular weight (5 kDa), which was expected to be below the diffusion limit of NPCs, was also excluded from the nucleus (Figure S4e), suggesting that the event of accumulation of a polymer at the spindle region was dependent on physicochemical properties, such as the existence of branching in the polymer structure.

We then compared the accumulation dynamics of dextrans with molecular weights of 3, 40, 70, and 150 k. Dextran (3 kDa) presented with continuous accumulation in the nuclear region throughout the cell cycle (Figure S4f and S4g), probably because the molecular weight was below the diffusion threshold of the nuclear pore complex. Other dextrans exhibited NEBD-dependent accumulation at the nascent spindle region (Figure S4b and Movie S4). As depicted in the time series of the normalized NI, dextrans accumulated only after NEBD (Figure S4g). The time series also did not demonstrate any marked difference in the timing of dextran accumulation. This result indicated that molecular weight was not a determinant factor for the accumulation order.

Although molecular weight was not deemed the determinant, it was notable that exogenous polymers showed an accumulation pattern similar to that shown by dynein and the regulatory proteins. Additionally, it was observed that the proteins, dynein and dynactin, mainly accumulated through the establishment of interaction with microtubules (Figures 3d and 3e). Thus, we examined whether the accumulation of dextran depended on the interaction with microtubules. The observation of dextran in the nocodazole-treated embryos showed that it continued to accumulate at the nascent spindle region

(Figure S4h), indicating that the accumulation of dextran was not dependent on microtubules such as LIS-1, NUD-2, and LIN-5 (Figures 3a-c). Furthermore, similar to LIN-5, dextrans showed a uniform distribution in the nascent spindle region (Figure S4i). Although the accumulation dynamics of dextrans shared several characteristics with dynein and the regulatory proteins, dextran did not present with accumulation before NEBD, as that observed for NUD-2, suggesting an additional requirement for such an accumulation pattern.

2. Pre-NEBD accumulation of NUD-2 is independent of NEBD

Among the proteins observed, NUD-2 showed a distinct accumulation pattern compared with the other proteins; accumulation started approximately 4 min before NEBD and the highest maximum normalized NI of approximately 4.5-fold was noted (Figures 2d and 4a). We termed this phenomenon "pre-NEBD accumulation" and investigated it comprehensively.

We observed that the initiation time of pre-NEBD accumulation was around the time of the pronuclear meeting. If the pre-NEBD accumulation is dependent on pronuclear meetings, it should occur only at the 1-cell stage because the pronuclear meeting is specific to the 1-cell stage. However, this was not the case. We found that pre-NEBD accumulation also occurred in the later stage embryos (2–16-cell stage; Figure S6a). Interestingly, as development proceeded, the degree of accumulation through pre-NEBD accumulation increased, while the final normalized NI after post-NEBD accumulation did not vary among the cell stages (Figures S6b-d). In contrast to the early embryos, in oocytes, NUD-2 did not accumulate to the nuclear region prior to the NEBD of the oocyte meiosis. The post-NEBD accumulation was observed for the oocyte meiosis (Figures S6e and S6f). Moreover, we found that NUD-2 localized at the nuclear membranes in all oocytes except the most proximal (-1) one (Figure S6e), in contrast to the early embryos. These results suggest that pre-NEBD accumulation is specific to mitotic division, whereas post-NEBD accumulation is universal to mitosis and meiosis.

We then investigated the relationship between pre-NEBD accumulation of NUD-2 and NEBD. We focused on a key aspect: was pre-NEBD accumulation coupled with NEBD? If pre-NEBD accumulation depends on NEBD, the timing of pre-NEBD accumulation between sperm- and oocyte-derived pronuclei will differ in the presence of nocodazole. Nocodazole treatment impairs pronuclear meeting, which in turn delays NEBD of the oocyte pronucleus due to the lack of signals from centrosomes attached to the sperm pronucleus[4–6]. When NEBD of oocyte pronucleus was delayed, there was no delay in the initiation time of pre-NEBD accumulation and it occurred at the same time as that of sperm pronucleus (Figures S6g and S6h). After reaching a value of approximately 1.3, the normalized NI of the oocyte pronuclei showed the achievement of a steady state for several minutes, while the normalized NI of the sperm pronucleus showed a transition to post-NEBD accumulation.

These results suggest that pre-NEBD accumulation is a distinct process from the post-NEBD accumulation and is regulated by factors independent of NEBD.

3. NUD-2 exhibits a distinct accumulation pathway from tubulin

Ran, a small GTPase protein, plays a central role in nuclear transport. A recent study revealed that Ran contributed to the accumulation of a tubulin chaperone in the nuclear region before NEBD in *Drosophila melanogaster*[7]. We have previously shown that RAN-1 is necessary for the post-NEBD accumulation of tubulin in *C. elegans* embryos[8]. We sought to ascertain whether Ran was involved in the pre-NEBD accumulation of NUD-2 by conducting knockdown experiments for *C. elegans* Ran, *ran-1*. In the *ran-1* (RNAi) embryos, we confirmed a reduction in cell size, nuclear size, and observed defects in mitosis (Figure S7a), as those previously described[9–11]. The defect in cytokinesis maintained the embryos in the 1-cell stage, although the nuclei underwent multiple divisions. Even under such conditions, we observed the cyclic accumulation of NUD-2 at the sites of histone signals (Figures S7a and S7b, and Movie S4). Such accumulation was not observed for tubulin[8] (Figures S7c and S7d). These results suggest that NUD-2 exhibits a different accumulation pathway from tubulin, whose post-NEBD accumulation is dependent on RAN-1[8].

To investigate the details of NUD-2 accumulation in the *ran-1* (RNAi) embryos, we analyzed the time series of the normalized NI. In *ran-1* (RNAi) embryos, we could not determine the timing of NEBD from the localization pattern of histones, and thus it was difficult to differentiate between pre-NEBD and post-NEBD accumulation events of NUD-2. As shown in Figure S7e, NUD-2 signals increased at an approximately constant rate. This increasing pattern was somewhat different from the unperturbed condition where we observed slower accumulation followed by a short constant phase before NEBD and faster accumulation after NEBD (Figure 4a). We considered that either pre- or post-NEBD accumulation was impaired by *ran-1* (RNAi). By comparing the rate of accumulation, we found that the accumulation rate under the *ran-1* (RNAi) condition was more similar to that under the unperturbed condition (Figure S7e). Furthermore, the maximum normalized NI of NUD-2 in the absence of RAN-1 was estimated to be 3.8 ± 1.6 (based on 7 increase events in 5 embryos), comparable to that of post-NEBD accumulations under the unperturbed condition (4.7 ± 0.6). These results suggest that Ran is necessary for pre-NEBD accumulation, but is not vital in the post-NEBD accumulation of NUD-2. This is in contrast to tubulin, where post-NEBD accumulation is impaired by *ran-1* (RNAi)[8]. These results suggest that the mechanism of post-NEBD accumulation is different between NUD-2 and tubulin.

Supplementary References

1. Tzur, Y. B. & Gruenbaum, Y. *Nuclear Envelope Breakdown and Reassembly in C. elegans: Evolutionary Aspects of Lamina Structure and Function*. (Landes Bioscience, 2013).
2. Galy, V., Mattaj, I. W. & Askjaer, P. Caenorhabditis elegans nucleoporins Nup93 and Nup205 determine the limit of nuclear pore complex size exclusion in vivo. *Mol. Biol. Cell* **14**, 5104–5115 (2003).
3. Updike, D. L., Hachey, S. J., Kreher, J. & Strome, S. P granules extend the nuclear pore complex environment in the C. elegans germ line. *J. Cell Biol.* **192**, 939–948 (2011).
4. Hachet, V., Canard, C. & Gönczy, P. Centrosomes promote timely mitotic entry in C. elegans embryos. *Dev. Cell* **12**, 531–541 (2007).
5. Portier, N. *et al.* A microtubule-independent role for centrosomes and aurora a in nuclear envelope breakdown. *Dev. Cell* **12**, 515–529 (2007).
6. Toya, M., Terasawa, M., Nagata, K., Iida, Y. & Sugimoto, A. A kinase-independent role for Aurora A in the assembly of mitotic spindle microtubules in Caenorhabditis elegans embryos. *Nat. Cell Biol.* **13**, 708–714 (2011).
7. Métivier, M. *et al.* Drosophila Tubulin-Specific Chaperone E Recruits Tubulin around Chromatin to Promote Mitotic Spindle Assembly. *Curr. Biol.* **31**, 684-695.e6 (2021).
8. Hayashi, H., Kimura, K. & Kimura, A. Localized accumulation of tubulin during semi-open mitosis in the Caenorhabditis elegans embryo. *Mol. Biol. Cell* **23**, 1688–1699 (2012).
9. Gönczy, P. *et al.* Functional genomic analysis of cell division in C. elegans using RNAi of genes on chromosome III. *Nature* **408**, 331–336 (2000).
10. Askjaer, P., Galy, V., Hannak, E. & Mattaj, I. W. Ran GTPase cycle and importins alpha and beta are essential for spindle formation and nuclear envelope assembly in living Caenorhabditis elegans embryos. *Mol. Biol. Cell* **13**, 4355–4370 (2002).
11. Bamba, C., Bobinnec, Y., Fukuda, M. & Nishida, E. The GTPase Ran regulates chromosome positioning and nuclear envelope assembly in vivo. *Curr. Biol.* **12**, 503–507 (2002).

Supplementary Figures

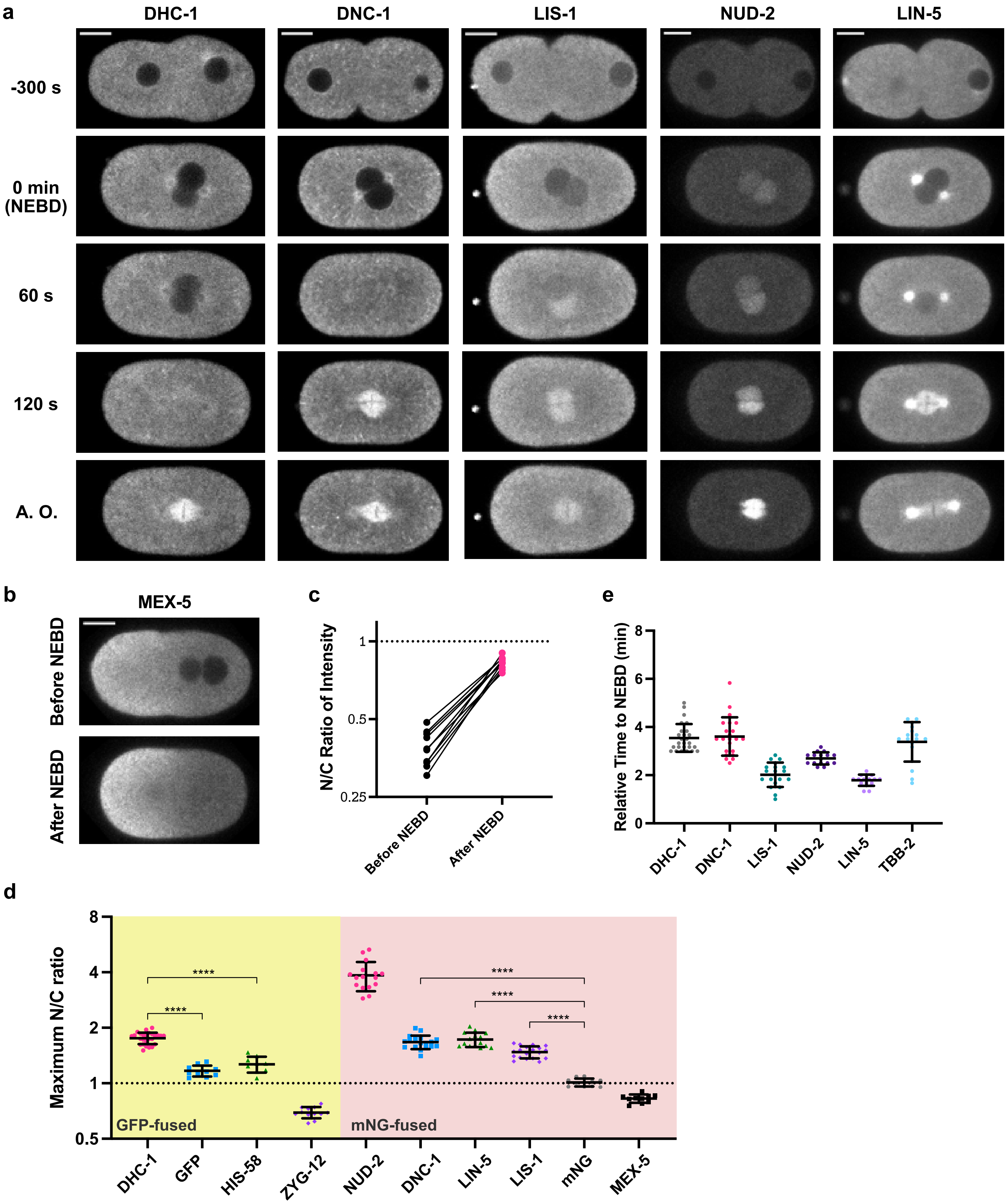

Figure S1. Accumulation of dynein, dynactin, LIS-1, NUD-2, and LIN-5.

(a) Typical single-plane time-lapse images showing the temporal dynamics of the proteins indicated above. The indicated times are relative to NEBD. “A. O.” denotes anaphase onset. (b) The images showing the spatiotemporal dynamics of mNG::MEX-5 expressed from the endogenous locus. (c) The graph showing the comparison of the N/C ratio of MEX-5 between before and after NEBD. The

number of pronuclei analyzed was 10 from 5 embryos. (d) Comparison of the maximum N/C ratio among DHC-1, GFP, ZYG-12, DNC-1, LIS-1, LIN-5, mNG, and MEX-5. The numbers of pronuclei analyzed are 26 from 14 embryos (dynein, DHC-1), 10 from 8 embryos (GFP), 9 from 6 embryos (histone, HIS-58), 12 from 6 embryos (ZYG-12), 16 from 8 embryos (NUD-2), 18 from 11 embryos (dynactin, DNC-1), 19 from 11 embryos (LIS-1), 15 from 10 embryos (LIN-5), and 11 from 6 embryos (mNG), and 10 from 5 embryos (MEX-5). Statistical significance was determined by Tukey's multiple comparison test in GFP-fused and mNG-fused proteins, separately (***: $p < 0.001$ and ****: $p < 0.0001$). The maximum N/C ratio of LIS-1 was significantly lower than that of other mNG-fused dynein-related proteins (DNC-1, NUD-2, and LIN-5), determined by Tukey's multiple comparison test ($p < 0.0001$). NUD-2 was excluded from the statistical test because it was apparently abundant. (e) The graph showing the time when the maximum NI was achieved. The mean and SD are indicated by the bars. The numbers of embryos analyzed are the same as (d). (a and b) All the scale bars indicate 10 μm. All the embryos in the images are aligned with the anterior side facing left.

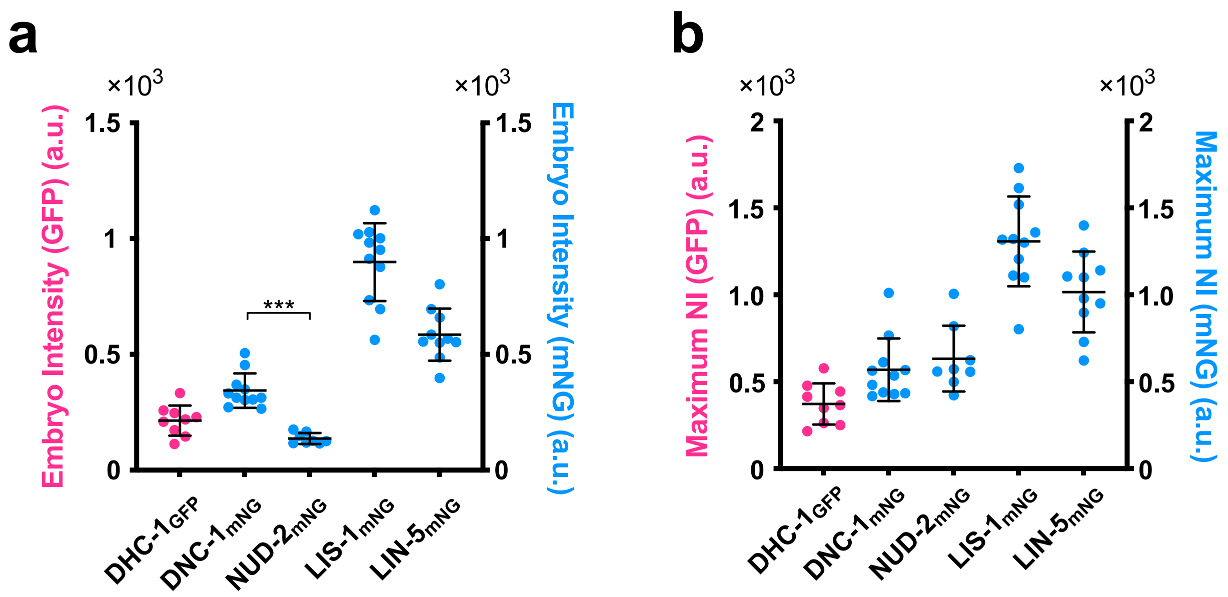

Figure S2. Quantification of endogenously-tagged dynein and the regulatory proteins. (a) Embryonic intensity (EI). (b) The maximum NI. Note that the fluorescent tags used for DHC-1 (GFP) and the other proteins (mNG) were different as indicated with different colors of the dots and axes. The statistical significance determined by Tukey's multiple comparison test were conducted among the mNG-fused proteins and the statistical significance between pairs of interest are shown (***: $p < 0.001$). The comparison was conducted on the mNG-fused proteins. The analyzed embryos are the same as in Figure 1. The bars indicate the mean and the SD.

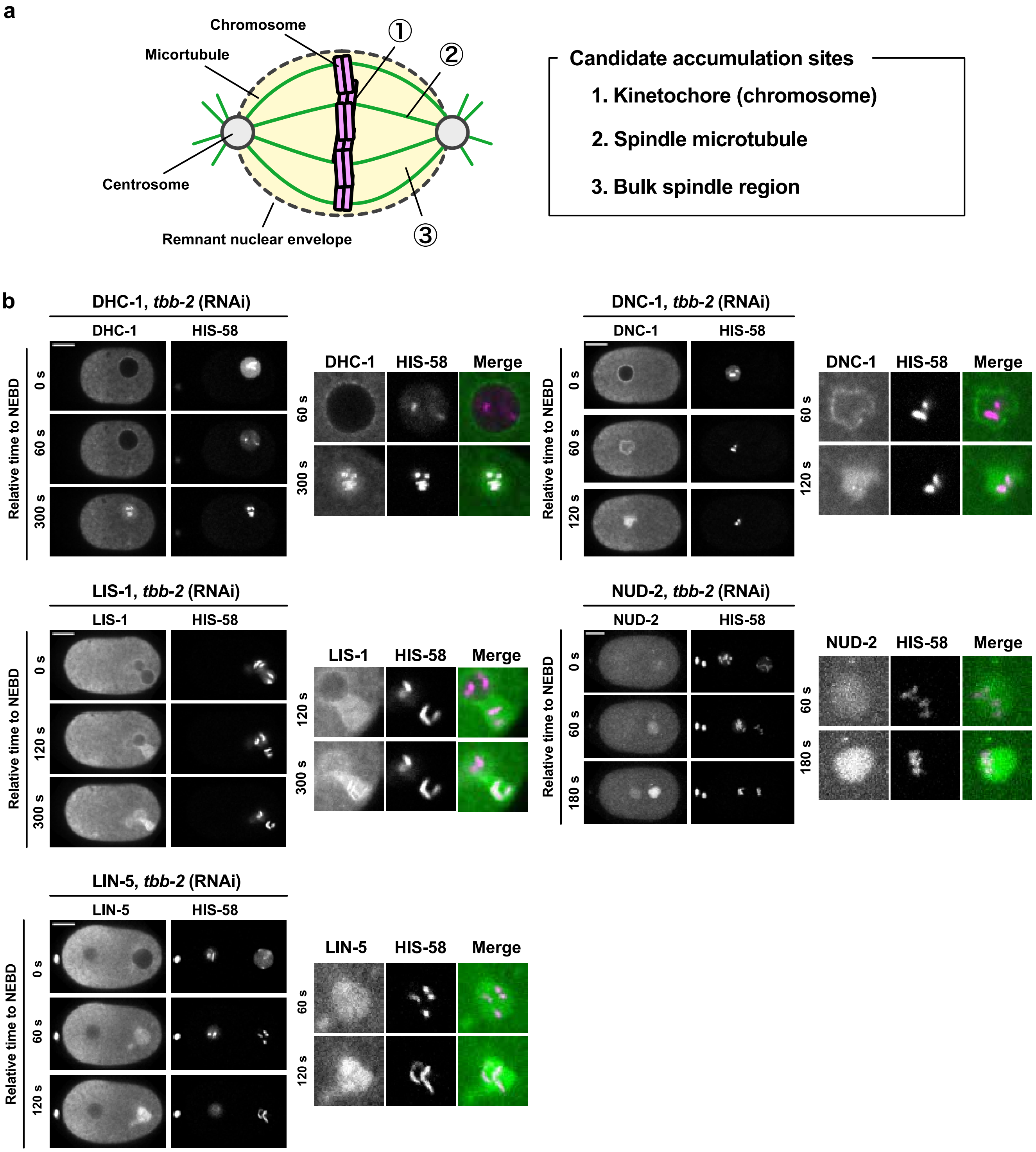

Figure S3. Variations in accumulation sites of dynein and its regulatory proteins.

(a) Schematic representation of accumulation site candidates. (b) Accumulation patterns in the *tbb-2* embryos. The spatial distribution of LIS-1, NUD-2, LIN-5, dynein (DHC-1), and dynactin (DNC-1) in *tbb-2* (RNAi) embryos is presented. Single-plane time-lapse images of whole embryos and a magnified image of the male pronuclei are shown. The left side of the image corresponds to the anterior. The scale bars indicate 10 μm.

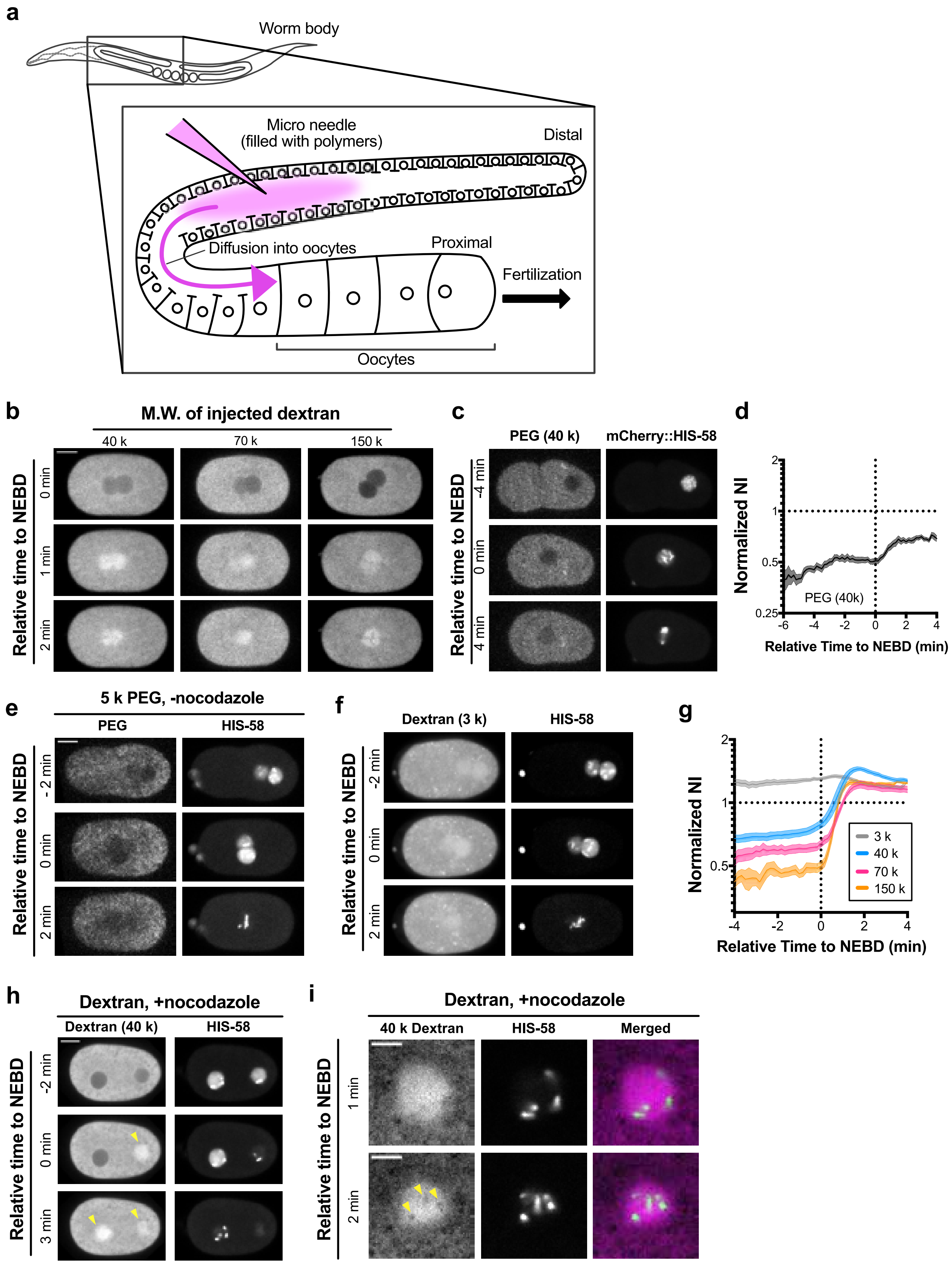

Figure S4. Observation of polymer incorporated through gonad injection. (a) Schematic representation of polymer injection experiments. (b) Typical single-plane time-lapse images showing the temporal dynamics of dextrans incorporated through the gonad injection. The molecular weight of

the injected dextran is indicated above. The left side of the image corresponds to the anterior. The scale bars indicate 10 μm. (c) Typical single-plane time-lapse images showing the temporal dynamics of mPEG (40 k). The left side of the image corresponds to the anterior. The scale bar indicates 10 μm. (d) Time series of the normalized NI of mPEG (40 k). The number of analyzed pronuclei was 14 from 10 embryos. Mean and SEM are shown. (e) Typical single-plane time-lapse images showing the accumulation pattern of PEG (5 k). The left side of the image corresponds to the anterior. The scale bars indicate 10 μm. (f) Typical single-plane time-lapse images showing the temporal dynamics of dextran (3 k). The left side of the image corresponds to the anterior. (g) Time series of the normalized NI of the injected dextrans (3 k, 40 k, 70 k, and 150 k). The numbers of pronuclei analyzed were 9 from 6 embryos (3 k), 7 from 4 embryos (40 k), 8 from 4 embryos (70 k), and 9 from 6 embryos (150 k). Mean and SEM are shown (h) Typical single-plane time-lapse images showing the accumulation of dextran (40 k) in the presence of 10 μg/mL nocodazole. The scale bar indicates 10 μm. The left side of the image corresponds to the anterior. (i) Magnified images showing the distribution of dextran (40 k) in the oocyte-derived pronucleus. The yellow arrowheads indicate the lack of dextran signal in the sites of histone signals. The scale bar indicates 5 μm.

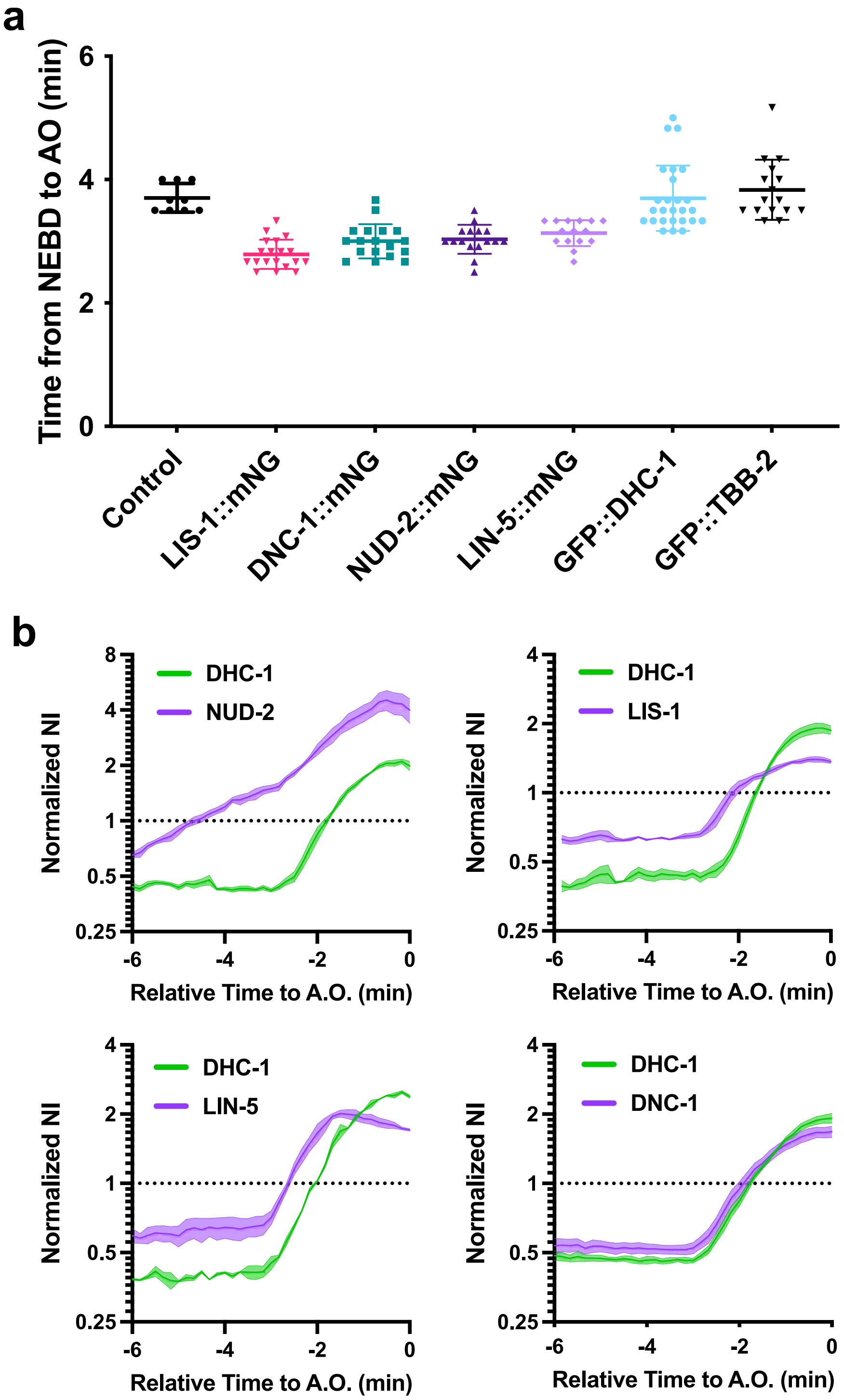

Figure S5 Temporal analysis and comparison of accumulations of dynein and the related proteins. (a) Time elapsed from NEBD to AO. The number of pronuclei analyzed was 9 from 5 embryos in the control experiment. For other conditions, the numbers of pronuclei analyzed are the same as in Figure 4a. The bars indicate the mean and the SD. (b) The time series of normalized NI in the oocyte-derived pronuclei obtained from the simultaneous observations, as shown in Figure 4d for the sperm-derived pronuclei. The numbers of pronuclei analyzed are 4 from 4 embryos (NUD-2), 4 from 4 embryos (LIS-1), 3 from 3 embryos (LIN-5), and 7 from 8 embryos (DNC-1). The mean and the SEM are shown.

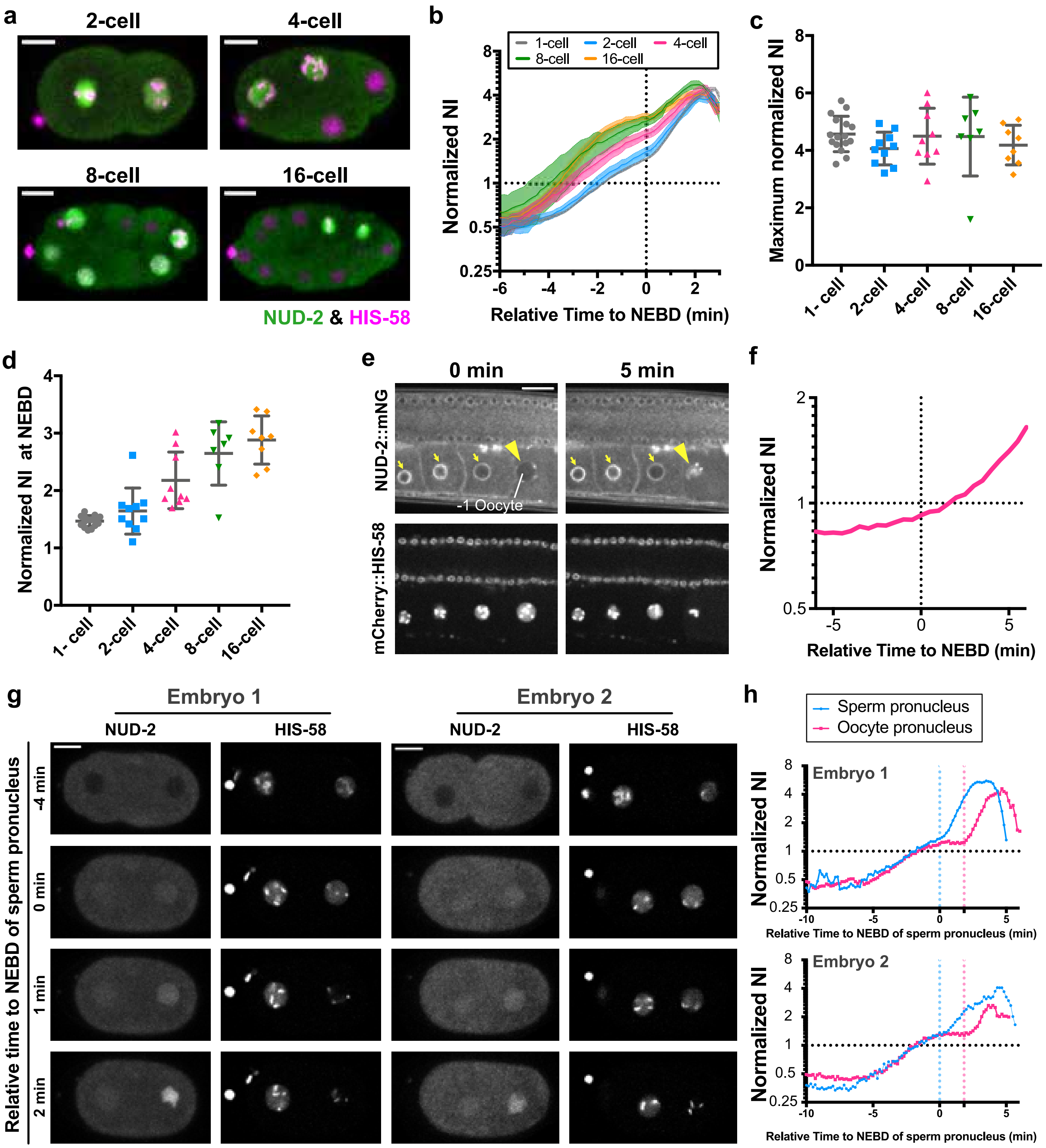

Figure S6. Accumulation patterns of endogenous NUD-2 in various contexts. (a) Single plane time-lapse images showing the signal of NUD-2::mNG (green) and mCherry::HIS-58 (magenta) in 2–16-cell stage embryos. The right side in the images corresponds to the anterior. The scale bars indicate 10 µm. (b) Time series of the normalized NI of NUD-2 in 2-cell (blue), 4-cell (red), 8-cell (green), and 16-cell (orange) embryos. For comparison, the time series of NUD-2 in 1-cell stage embryos is shown by using the gray line, which indicates the same data as indicated in Figure 4a. (c) Maximum normalized NI in 1–16-cell stage embryos. (d) The normalized NI measured at the time of NEBD. (e) Typical single plane time-lapse images depicting NUD-2 in the germline of an adult worm. The

yellow arrowheads denote the nucleus of the -1 oocyte, and the yellow arrows indicate the NUD-2 localizations at nuclear membranes. The times relative to NEBD of the -1 oocyte are indicated above. The scale bar indicates 20 μm. (f) Time series of the normalized NI of NUD-2 in the -1 oocyte shown in (e). (g) Typical single-plane time-lapse images showing the temporal dynamics of NUD-2 in the presence of 10 μg/mL nocodazole. The scale bars indicate 10 μm. (h) Time series of normalized NI of NUD-2 in the nocodazole-treated embryos. The normalized NI in sperm and oocyte pronuclei are depicted by using the blue lines and the magenta lines. The vertical dashed lines indicate the initiation of NEBD of pronuclei.

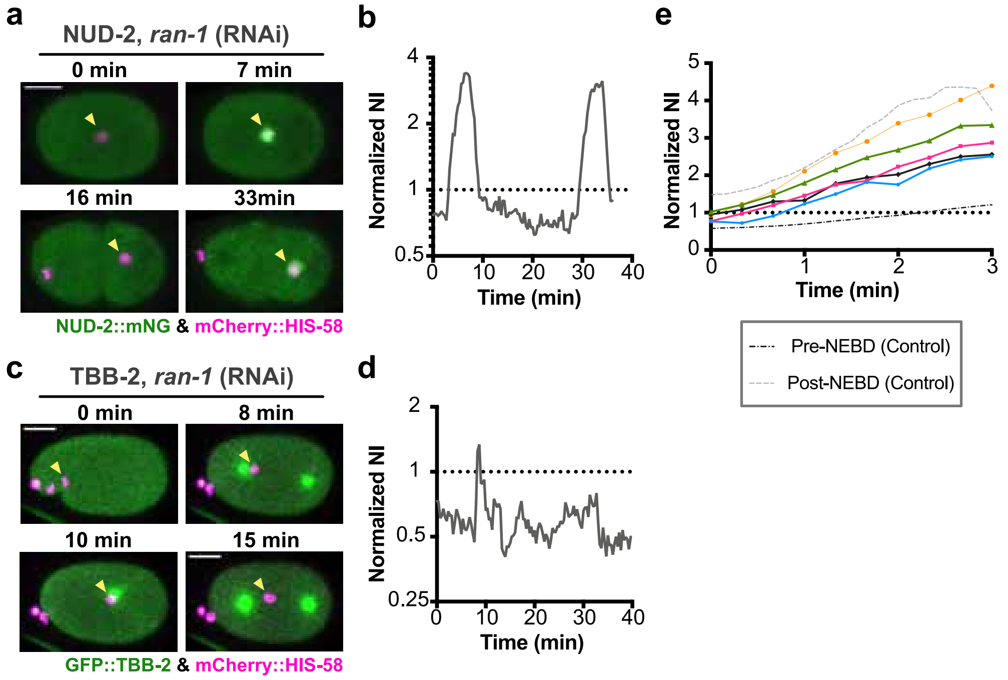

Figure S7. Accumulation of NUD-2 exhibits a distinct molecular dependency from tubulin. (a) Maximum projection images showing the temporal dynamics of NUD-2 in the *ran-1* (RNAi) embryo. Under *ran-1* (RNAi) conditions, the sizes of embryo and nucleus reduced, and the defect in cytokinesis was observed. Although it was difficult to detect the precise timing of NEBD, the cyclic increase in NUD-2 signals was confirmed. The left side of the image corresponds to the anterior. The scale bar indicates 10 μm. (b) Time series of normalized NI of NUD-2 in the *ran-1* (RNAi) embryos shown in (a). The intensity of NUD-2 in the region indicated by using the yellow arrowheads was measured. The origin of time was set to the initial time of the observation. Each peak seemed to demonstrate a rapid single-phase increase from the normalized NI below 1. (c) Maximum projection images showing the temporal dynamics of TBB-2 (tubulin) in the *ran-1* (RNAi) embryo. The left side of the image corresponds to the anterior. (d) Time series of normalized NI of TBB-2 in the *ran-1* (RNAi) embryos shown in (c). The intensity of NUD-2 in the region indicated by using the yellow arrowheads was measured. The origin of time was set to the initial time of the observation. (e) Comparison of temporal dynamics of the normalized NI between the unperturbed condition and *ran-1* (RNAi) conditions. The time series data of normalized NI during the rapid increase phase in *ran-1* (RNAi) embryos are shown by using the colored lines, while the black line and the gray line show the time series data of pre- and post-NEBD accumulations, respectively. The origin of time was set as the initial time of each accumulation, not the timing of NEBD because it was difficult to detect NEBD in the *ran-1* (RNAi) embryos.

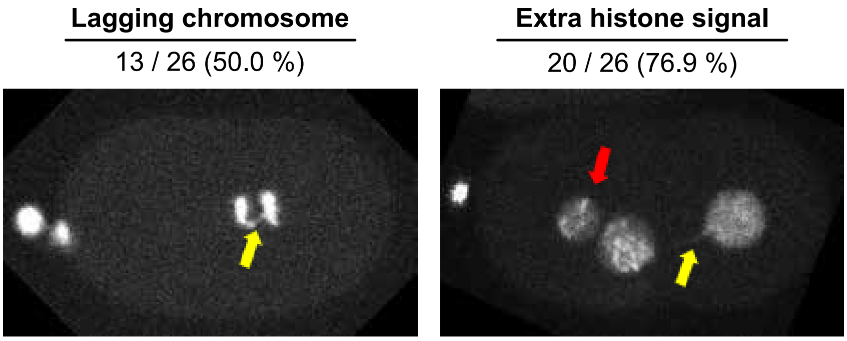

Figure S8. Abnormalities in chromosome dynamics in NUD-2 depleted embryos.

The images show the histone signal in the *C. elegans nud-2* (RNAi) embryos around 1st mitotic division. The yellow and red arrows indicate the lagging chromosomes and extra histone signal, respectively. The left sides of the images correspond to the anterior.

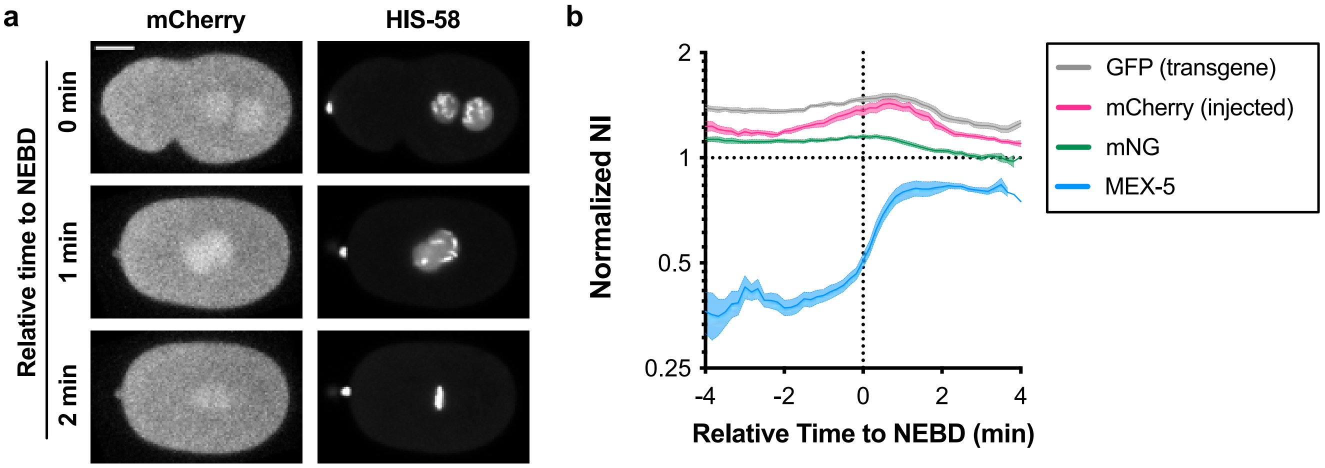

Figure S9. Temporal dynamics of injected mCherry. (a) Typical single-plane time-lapse images showing the temporal dynamics of SBP-mCherry incorporated into the early embryo. The right side of the image corresponds to the anterior. The scale bar indicates 10 µm. (b) Time series of the normalized NI of injected SBP-mCherry, transgenic GFP, mNG, and MEX-5. The numbers of pronuclei analyzed were 10 from 6 embryos (mCherry), 10 from 8 embryos (GFP), 12 from 6 embryos, and 10 from 5 embryos. Mean and SEM are shown.

Table S1. The worm strains used in this study

	Strain	Genotype	Comment	Figure	Source	Reference
**1**	WH223	ojIs9 [zyg-12(all)::GFP + unc-119(+)].		Figure 1<br>Figure S1	CGC	Malone, 2003
**2**	SV1619	dhc-1(he250[mCherry::dhc-1]) I.		-	CGC	Schmidt, 2017
**3**	LP373	mex-5(cp125[mNG-C1^3xFlag::mex-5]) IV.		Figure 1<br>Figure S1<br>Figure S9	CGC	Dickinson, 2017
**4**	LP439	nud-2(cp170[nud-2::mNG-C1^3xFlag]) I.		-	CGC	Heppert, 2018
**5**	LP563	dnc-1(cp271[dnc::mNG-C1^3xFlag]) IV.		-	CGC	Heppert, 2018
**6**	LP585	lin-5(cp288[lin-5::mNG-C1^3xFlag]) II.		-	CGC	Heppert, 2018
**7**	LP591	lis-1(cp294[lis-1::mNG-C1^3xFlag]) III.		-	CGC	Heppert, 2018
**8**	BN711	unc-119 (ed3);; bqSi711 [mex-5p::FLP::SL2::mNG + unc-119(+)] IV		Figure 1<br>Figure S1<br>Figure S9	CGC	Macias-Leon & Askjaer, 2018
**9**	CAL0234	ruIs32 [pie-1p::GFP::H2B + unc-119(+)] III.		Figure 6<br>Figure S4<br>Figure S9		Arai, 2017
**10**	CAL0361	unc-119(ed3) III; [unc-119(+); pie-1p::gfp]		-		Hayashi, 2012
**11**	CAL0491	unc-119(ed3) III; ltIs37[pAA64; pie-1::mCherry::HIS-58; unc-119(+)]; ruIs57[unc-119(+); pie-1p::gfp::tubulin]		Figure 1<br>Figure 4<br>Figure S7		Hayashi, 2012
**12**	CAL0941	unc-119 (ed3); wjIs108[unc-119: pie-1 5' : mCherry-his-58: pie-1 3']		Figure S4<br>Figure S8		This work
**13**	CAL2221	dhc-1(hsGFP::dhc-1) I.		-		This work
**14**	CAL2261	dnc-1(cp271[dnc::mNG-C1^3xFlag]) IV. : dhc-1(he250[mCherry::dhc-1]) I.	SV1619 x LP563	Figure 4<br>Figure S5		This work
**15**	CAL2271	lis-1(cp294[lis-1::mNG-C1^3xFlag]) III. : dhc-1(he250[mCherry::dhc-1]) I.	SV1619 x LP591	Figure 4<br>Figure S5		This work
**16**	CAL2281	lin-5(cp288[lin-5::mNG-C1^3xFlag]) II. : dhc-1(he250[mCherry::dhc-1]) I.	SV1619 x LP585	Figure 4<br>Figure S5		This work
**17**	CAL2291	nud-2(cp170[nud-2::mNG-C1^3xFlag]) I.: dhc-1(he250[mCherry::dhc-1]) I.	SV1619 x LP439	Figure 4<br>Figure S5		This work
**18**	CAL2302	nud-2(cp170[nud-2::mNG-C1^3xFlag]) I.: unc-119 (ed3); wjIs108[unc-119: pie-1 5' : mCherry-his-58: pie-1 3']	LP439 x CAL0941	Figure 1<br>Figure 2<br>Figure 3<br>Figure 4<br>Figure 5<br>Figure 6<br>Figure S1<br>Figure S2<br>Figure S3<br>Figure S5<br>Figure S6<br>Figure S7		This work
**19**	CAL2311	dnc-1(cp271[dnc::mNG-C1^3xFlag]) IV. : unc-119 (ed3); wjIs108[unc-119: pie-1 5' : mCherry-his-58: pie-1 3']	LP563 x CAL0941	Figure 1<br>Figure 2<br>Figure 3<br>Figure 4<br>Figure 5<br>Figure S1<br>Figure S2<br>Figure S3		This work

**20**	CAL2331	lin-5(cp288[lin-5::mNG-C1^3xFlag]) II. : unc-119 (ed3); wjIs108[unc-119: pie-1 5' : mCherry-his-58: pie-1 3']	LP585 x CAL0941	Figure 1<br>Figure 2<br>Figure 3<br>Figure 4<br>Figure 5<br>Figure S1<br>Figure S2<br>Figure S3<br>Figure S5	This work
**21**	CAL2341	lis-1(cp294[lis-1::mNG-C1^3xFlag]) III. : unc-119 (ed3); wjIs108[unc-119: pie-1 5' : mCherry-his-58: pie-1 3']	LP591 x CAL0941	Figure 1<br>Figure 2<br>Figure 3<br>Figure 4<br>Figure 5<br>Figure S1<br>Figure S2<br>Figure S3<br>Figure S5	This work
**22**	CAL2391	dhc-1(hsGFP::dhc-1) I. : unc-119 (ed3); wjIs108[unc-119: pie-1 5' : mCherry-his-58: pie-1 3']	CAL2221 x CAL0941	Figure 1<br>Figure 2<br>Figure 3<br>Figure 4<br>Figure 5<br>Figure S1<br>Figure S2<br>Figure S3<br>Figure S5	This work
**23**	CAL2461	unc-119 (ed3); wjIs108[unc-119: pie-1 5' : mCherry-his-58: pie-1 3'], unc-119(ed3) III; [unc-119(+); pie-1p::gfp]	CAL0941 x CAL0361	Figure 1<br>Figure S9	This work

Supplementary Movies

Movie S1. Accumulation dynamics of dynein, dynactin, LIS-1, NUD-2, and LIN-5. 100 × Real-Time. Scale bar: 10 µm.

Movie S2. Accumulation dynamics of dynein, dynactin, LIS-1, NUD-2, and LIN-5 in the presence of 10 µg/mL nocodazole. 100 × Real-Time. Scale bar: 10 µm.

Movie S3. Accumulation dynamics of dextrans (left) and HIS-58 (right). The molecular weights of dextran have been depicted in the movie. 100 × Real-Time.

Movie S4. Accumulation dynamics of NUD-2 and TBB-2 in *ran-1* (RNAi) embryos. 200 × Real-Time. Scale bar: 10 µm.

Movie S5. Accumulation dynamics of NUD-2 fragments. 100 × Real-Time. Scale bars: 10 µm.
